# Genome-wide association scan and transcriptome analysis reveal candidate genes for waterlogging tolerance in cultivated barley

**DOI:** 10.3389/fpls.2022.1048939

**Published:** 2022-12-15

**Authors:** Haiye Luan, Changyu Chen, Ju Yang, Hailong Qiao, Hongtao Li, Shufeng Li, Junyi Zheng, Huiquan Shen, Xiao Xu, Jun Wang

**Affiliations:** ^1^ College of Marine and Biological Engineering, Yancheng Teachers University, Yancheng, Jiangsu, China; ^2^ Institute of Agricultural Science in Jiangsu Coastal Areas, Yancheng, China; ^3^ Lianyungang academy of agricultural sciences, Lianyungang, China; ^4^ Jiangsu Provincial Key Laboratory of Coastal Wetland Bioresources and Environmental Protection, Yancheng, Jiangsu, China

**Keywords:** barley, waterlogging, genome-wide association scan, RNA-seq, candidate genes

## Abstract

Waterlogging is the primary abiotic factor that destabilizes the yield and quality of barley (*Hordeum vulgare* L.). However, the genetic basis of waterlogging tolerance remains poorly understood. In this study, we conducted a genome-wide association study (GWAS) by involving 106,131 single-nucleotide polymorphisms (SNPs) with a waterlogging score (WLS) of 250 barley accessions in two years. Out of 72 SNPs that were found to be associated with WLS, 34 were detected in at least two environments. We further performed the transcriptome analysis in root samples from TX9425 (waterlogging tolerant) and Franklin (waterlogging sensitive), resulting in the identification of 5,693 and 8,462 differentially expressed genes (DEGs) in these genotypes, respectively. The identified DEGs included various transcription factor (TF) genes, primarily including AP2/ERF, bZIP and MYB. By combining GWAS and RNA-seq, we identified 27 candidate genes associated with waterlogging, of which three TFs (*HvDnaJ*, *HvMADS* and *HvERF1*) were detected in multiple treatments. Moreover, by overexpressing barley *HvERF1* in *Arabidopsis*, the transgenic lines were detected with enhanced waterlogging tolerance. Altogether, our results provide new insights into the genetic mechanisms of waterlogging, which have implications in the molecular breeding of waterlogging-tolerant barley varieties.

## Introduction

Waterlogging is one of the major abiotic stresses that limits crop production and affects 16% of the global land area (Setter and Waters, 2003). As a result of global climate change, extreme weather events have become more frequent and severe in crop cultivated areas (Donat et al., 2016). Waterlogging is caused by high rain, irrigation practices and/or poor soil drainage, which results in anoxic soils and severe hypoxia in crop roots (Bailey-Serres et al., 2012). Waterlogging has severely limited the production of wheat and barley in the Yangtze River Plain of China. Furthermore, winter wheat grain yield was reported to be as low as 4978.5 kg ha^-1^ or zero in years with extreme precipitation (Ding et al., 2020; He et al., 2020).

Barley (*Hordeum vulgare* L.) is the fourth cereal crop worldwide and is primarily used for animal feed, malting and brewing (Pegler et al., 2018). Compared with other crop species, barley is more sensitive to waterlogging stress. Waterlogging causes a reduction in shoot and root growth, leaf area, and biomass and eventually leads to a reduction in crop yield (Ciancio et al., 2021). Barley, as with other plants, has evolved with diverse morpho-physiological, biochemical, transcriptional and metabolic strategies to overcome waterlogging stresses, such as the formation of adventitious roots, aerenchyma in shoots, plant hormones and reactive oxygen species (ROS) detoxification (Zhang et al., 2016; Luan et al., 2018a; Gill et al., 2019). Plant waterlogging tolerance is a complex trait, and the underlying mechanisms are still poorly understood.

The selection of waterlogging-tolerant varieties is an effective strategy for increasing barley yield. However, waterlogging tolerance is a complex trait controlled by several genes (Borrego et al., 2021). In general, marker-assisted selection (MAS) is a high-efficiency and economical approach that can overcome the inefficiencies of traditional phenotyping breeding. Recently, numerous quantitative trait loci (QTL) that are involved in waterlogging tolerance in barley, including leaf chlorosis, plant survival, plant biomass reduction, chlorophyll fluorescence, root porosity, and aerenchyma development, have been identified by linkage analysis of doubled haploid (DH) (Zhou, 2011; Zhou et al., 2012; Broughton et al., 2015; Zhang et al., 2016; Zhang et al., 2017). However, QTL mapping for targeted traits is dependent on the polymorphisms between the parents and the population size (Wang et al., 2020).

A genome-wide association study (GWAS) is an effective approach to identify genomic regions associated with specific variants of complex traits, which could dissect more alleles compared with linkage analysis. Recently, GWAS has been widely used to detect important candidate genes associated with yield, quality, salt stress, and drought stress (Reig-Valiente et al., 2018; Luo et al., 2021; Hao et al., 2022; Wu et al., 2022). In barley, many functional loci associated with agronomic traits (Xu et al., 2018), salt stress tolerance (Mwando et al., 2020), drought stress tolerance (Tarawneh et al., 2020), grain quality (Jia et al., 2021) and disease resistance (Pan et al., 2022) have been identified by GWAS. Borrego et al. (2021) were the first to identify 51 significant markers associated with barley waterlogging tolerance under controlled field conditions. RNA sequencing (RNA-seq) is a valuable tool for identifying candidate genes and regulation pathways, and has been used widely in plants response to waterlogging stress (Borrego et al., 2020; Sharmin et al., 2020; Chen et al., 2021). Combined GWAS and RNA-seq have been shown to identify candidate genes and provide molecular makers for MAS more efficiently. For example, Zhao et al. (2021) detected eight candidate genes and developed KASP markers for verticillium wilt resistance in cotton by combining GWAS and RNA-seq. Jia et al. (2020) identified six candidate genes of grain drying rate in maize with GWAS, and one of the candidate genes was verified by transcriptomic data.

In this study, we first performed a GWAS analysis of waterlogging-related traits among 250 barley accessions grown across four different periods in two years. Next, we performed RNA-seq analysis to identify the genes involved in waterlogging tolerance in barley. Through the combination of GWAS and RNA-seq analysis, we identified candidate genes related to waterlogging tolerance in barley. Finally, we validated candidate genes with qRT-PCR and transgenic *Arabidopsis*. The results may provide helpful information to better understand the molecular mechanism of waterlogging tolerance in barley.

## Materials and methods

### Plant materials and phenotypic analysis

**Figure 1:**
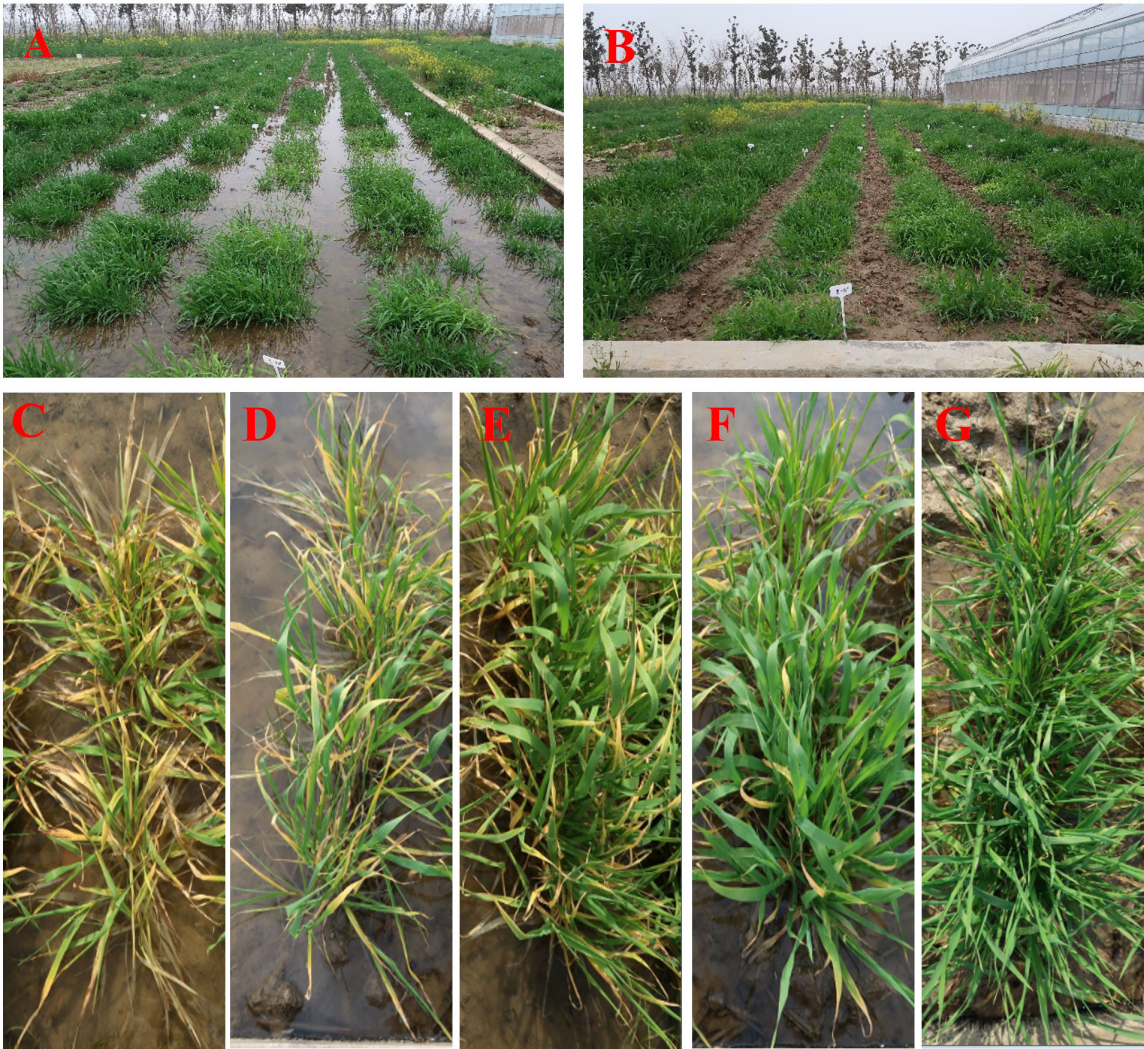
A cement pool experiment used to screen 250 barley lines for waterlogging tolerance **(A, B)**. **(A)** Waterlogging treatment. **(B)** Control. Barley lines with different waterlogging tolerance scores **(C–G)**. **(C)** 1; **(D)** 2; **(E)** 3; **(F)** 4; **(G)** 5.

In total, 250 barley accessions including 172 genotypes from China and 78 exotic lines, from 19 countries, were used in the association mapping of waterlogging tolerance at the tillering stage (**Supplementary Table 1**). These accessions were composed of 148 two-rowed and 102 six-rowed barley. The plants were cultured in a cement pool containing soil and subjected to waterlogging at the tillering stage (keeping the water level above the soil surface). Seeds were sown with a randomized block design over three consecutive years (2018-2020) and three replicates were used in both waterlogging and controls. Each pool contained 250 rows, with 10 plants per row, 3 cm between plants within each row and 30 cm between rows. The waterlogging score (WLS) was assessed based on leaf chlorosis and plant survival. Durative waterlogging was kept for four weeks, and then, plants were scored from 1 (susceptible) to 5 (tolerant) (1, leaf chlorosis of plants ≥80%; 2, leaf chlorosis of plants 60-80%; 3, leaf chlorosis of plants 40-60%; 4, leaf chlorosis of plants 20-40%; 5, leaf chlorosis of plants ≤20%) (**Figure 1**). WLS-1 represents waterlogging for 2 weeks, WLS-2 represents waterlogging for 3 weeks, WLS-3 represents waterlogging for 3 weeks, WLS-4 represents 2 weeks after drained water.

### Genome-wide association scanning

Genomic DNAs were extracted from young leaves. DNA degradation and contamination were checked on 1% agarose gels, and DNA purity was checked using the NanoPhotometer^®^ spectrophotometer (IMPLEN, CA, USA). DNA library construction and sequencing were performed by Novogene Bioinformatics Technology (Beijing, China). Single-nucleotide polymorphism (SNPs) annotation was performed according to the barley cultivar Morex (Mascher et al., 2017) (http://plants.ensembl.org/Hordeum_vulgare/Info/Annotation/#assembly) using the package ANNOVAR (Version: 2013-05-20) (Wang et al., 2010). To clarify the phylogenetic relationship from a genome-wide perspective, an individual-based neighbor-joining tree was constructed based on the p-distance using the software TreeBest (http://treesoft.sourceforge.net/treebest.shtml). The software MEGA6.0 (http://www.megasoftware.net/) was used for visualizing the phylogenetic trees. SNP calling was implemented in the package SAMtools (Li et al., 2009). Based on reads from each individual’s genomic location, genotype likelihoods were calculated, and the allele frequencies were calculated using Bayesian inference. After filtering with minor allele frequency (MAF) ≥ 0.05, SNP call rate≥ 0.95, and missing rate≤ 0.01, 106,131 high-quality SNPs were used in our GWAS for waterlogging-tolerant traits. The association analysis was conducted using the GEMMA (genome-wide efficient mixed-model association) (Zhou and Stephens, 2012) software package by incorporating the population analysis with the relative kinship matrix. Significant SNP markers associated with the target traits were identified according to the standard of log10 P > 4.0 (Tu et al., 2021). The candidate genes were selected within a 100 kb upstream and 100 kb downstream region delimited by each significant SNP (Tu et al., 2021).

### RNA-seq and transcription analysis

Based on the waterlogging score of the 250 genotypes and previous study (Zhou, 2011), the tolerant cultivar Taixing 9425 and the sensitive cultivar Franklin were used to RNA-seq analysis under waterlogging stress. The roots of samples were collected after waterlogging treatment for 72 h, and control without waterlogging. Each treatment was processed with three biological replicates. Total RNA was extracted using the Plant RNA Purification Kit (Tiangen, Beijing, China). Twelve RNA-seq libraries (two accessions × two treatment × three biological replicates) were constructed by Novogene Bioinformatics Technology (Beijing, China) and sequenced by an Illumina HiSeq 2500 platform (Illumina Inc., San Diego, CA, USA). The data presented in the study are deposited in the NCBI SRA repository, accession number PRJNA889532. Initially, raw fastq reads were processed through custom perl scripts. Then, raw data was cleaned by removing adapter, ploy-N, and low-quality reads. In addition to the Q20, Q30 and GC content in the clean data were calculated. High-quality clean data was used in all downstream analyses. A transcript abundance estimate for each gene was calculated using FPKM value. And the DEGs were further filtered with P value ≤ 0.05 and normalized fold change (FPKM in the waterlogging group/control group) ≥ 1 (Luan et al., 2016).

### Quantitative real-time RT-PCR

To confirm the reality of candidate genes screened from the analysis of GWAS and RNA-seq. 8 candidate genes were selected to further validate by quantitative (qRT-PCR). The method of qRT-PCR was described as previous report (Luan et al., 2018a). cDNA was initially synthesized using Random Primer 6 and M-MLV reverse transcriptase (Takara, Tokyo, Japan). The specific primers used for target were designed using the Primer Premier 5.0. All the primers are listed in **Supplementary Table S8**. The Hvactin gene was used as the internal control. A ViiA™ 7 Real-Time PCR System (Carlsbad City, CA, USA) was used for quantitative real-time PCR. Target genes’ relative expression levels were determined as 2-ΔCt. Three biological replicates and three technical repeats were performed in all the qRT-PCR experiments.

### Candidate gene validation by transgenic Arabidopsis

To further verify the candidate gene, transgenic Arabidopsis overexpressing HvERF1 were generated by floral dipping. The detailed design and methods have been previously described (Luan et al., 2020). The Gateway technology (Invitrogen, USA) was used to constructed the expression vectors. Through the floral dipping method, recombinant vectors were transferred into Arabidopsis (Columbia) using the Agrobacterium tumefaciens strain GV3101 (Clough and Bent, 1998). Homozygous Arabidopsis lines containing single-site transgene insertions were identified and maintained in growth until T3 generation. Further genetic analysis was performed using the homozygous T3 generation. Five-week-old Arabidopsis plants (T3 lines) were used for waterlogging treatment. The control plants were kept in normal conditions with regular watering. After the treatment of two weeks, the phenotypic traits were observed and recorded. For the analysis of gene expression related to waterlogging, shoots were collected at different times (0d, 3d, 6d, 9d) after waterlogging treatment. The internal control was conducted using Arabidopsis actin. The list of the primes used in this experiment can be found in **Supplementary Table S8**.

## Results

### Analysis of phenotypic variation

The WLS values were measured in 250 barley genotypes at different stages, and the results are presented in **Table 1** and **Table S2**. The plant growth was significantly impeded by waterlogging stress. The mean values of WLSs were 2.27, 2.74, 3.07 and 2.22 in 2019. The mean values were found to be higher along with increasing waterlogging duration, while the value decreased under two weeks after draining water. The variation trend of the mean was basically similar between 2019 and 2020. Under waterlogging conditions, the CVs (coefficient variations) ranged from 23.86 in 2019 WLS-4 to 42.70 in 2020 WLS-1.

**Table 1:** Phenotypic variation of barley plants under waterlogging stress.

Trait	2019				2020			
	WLS-1	WLS-2	WLS-3	WLS-4	WLS-1	WLS-2	WLS-3	WLS-4
Min	1.00	1.00	2.00	1.00	1.00	1.00	1.00	1.00
Max	4.00	5.00	5.00	5.00	5.00	5.00	5.00	5.00
Mean	2.27	2.74	3.07	2.22	2.19	2.92	3.43	2.29
SD	0.75	0.77	0.73	0.53	0.94	0.89	0.90	0.71
CV(%)	32.86	28.00	23.95	23.86	42.70	30.50	26.20	31.03

WL, waterlogging score.

The correlation analysis among different waterlogging treatment stages is shown in **Table 2**. WSL-1 in 2019 and2020 showed the highest consistency across WSL-4 in two years, with a correlation coefficient r^2^ = 0.747 and 0.819, respectively. However, 2019 WLS-1 showed a weak correlation with 2020 WLS-2 and 2020 WLS-3 (correlation coefficients were 0.064 and -0.062, respectively). Furthermore, 2019 WLS-4 showed less correlation with 2020 WLS-2 and 2020 WLS-3 (correlation coefficients were 0.086 and -0.042, respectively). These results suggeste that waterlogging score is a highly heritable trait that may be suitable for GWAS.

**Table 2:** Correlation analysis of waterlogging score at different stages.

Traits	2019WLS-1	2019WLS-2	2019WLS-3	2019WLS-4	2020WLS-1	2020WLS-2	2020WLS-3	2020WLS-4
2019WLS-1	1							
2019WLS-2	0.4**	1						
2019WLS-3	0.142**	0.539**	1					
2019WLS-4	0.747**	0.426**	0.139**	1				
2020WLS-1	0.177**	0.235**	0.286**	0.215**	1			
2020WLS-2	0.064	0.272**	0.254**	0.086	0.558**	1		
2020WLS-3	-0.062	0.191**	0.335**	-0.042	0.437**	0.705**	1	
2020WLS-4	0.228**	0.295**	0.327**	0.281**	0.819**	0.542**	0.404**	1

* and ** indicated the signficant correlation at P < 0.05 and P < 0.01

### Genome-wide association study of waterlogging stress tolerance

Based on the sequencing results, we obtained 6,536,895 SNPs distributed across 7 barley chromosomes. After quality control, 106,131 SNP loci were used for subsequent GWAS analyses (**Figure S1**). The population structure analysis suggested that the population could be classified into two groups (**Figure S2**). Subpopulation 1 primarily included 58 genotypes composed of local varieties in China, while subpopulation 2 included 192 cultivars from different countries (**Figure S2**; **Table S1**).

**Figure 2:**
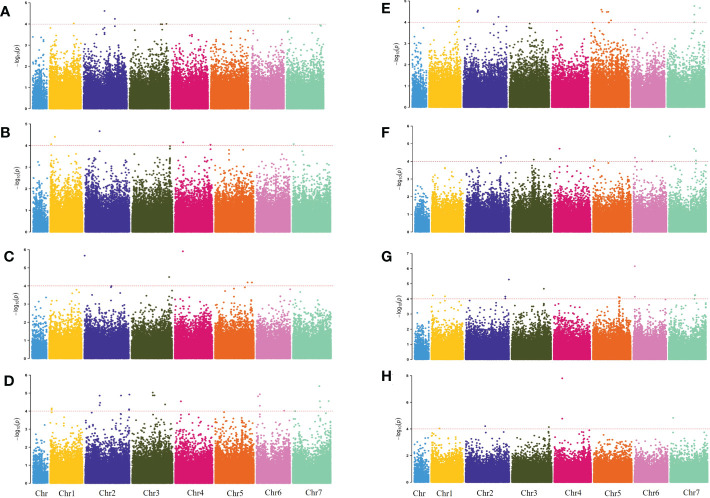
Manhattan plots for 2019 WLS-1, 2019 Q34 WLS-2, 2019 WLS-3, 2019 WLS-4, 2020 WLS-1, 2020 WLS-2, 2020 WLS-3, 2020 WLS-4 were shown in **(A–H)**, respectively. The x-axis shows SNP loci along the seven barley chromosomes. The horizontal red line shows the genome-wide significance threshold P-value of –log10 (P-value) value of 4.0. GWAS was performed using the MLM (Q + K) model.

The genome-wide association scanning was conducted by using the GLM and MLM algorithms to identify significant SNPs associated with waterlogging stress. A total of 356 SNPs were associated with waterlogging tolerance when GLM was performed (**Figure S3**; **Table S3**). While with MLM analysis, only 72 significant SNPs were found and all these associations were common in the GLM (**Figure 2**; **Table S4**). The MLM model was more efficient in reducing false positive associations. Therefore, significant SNPs finalized based only on MLM were presented here. Among these, 34 were detected in at least two environments (two years and four development stages). As false positives were always caused by a single environment, four overlapping SNPs (chr2H-250021530, chr2H-258433925, chr4H-138201763, chr6H-26353758) in three different stages were defined as significant, which were mainly anchored in chromosomes 2, 4 and 6. Only one SNP (chr7H-478156203) in four different stages was defined in chromosome 7 (**Table 3**).

**Table 3:** Significant SNPs associated with waterlogging identified across two or more environments.

Traits	Marker Name	Chr	POS	REF	ALT	*p* value	Annotation
2019 WSL-1	chr7H-51003579	7	51003579	G	A	5.50E-05	Vesicle-associated membrane protein 713
2019 WSL-2	chr2H-249486624	2	249486624	T	A	2.17E-05	MATE efflux family protein
	chr4H-138201763	4	138201763	A	C	7.17E-05	Nuclear pore complex protein Nup98-Nup96
2019 WSL-3	chr3H-649787421	3	649787421	C	T	3.23E-05	MADS-box transcription factor family protein
	chr4H-138201763	4	138201763	A	C	1.24E-06	Nuclear pore complex protein Nup98-Nup96
	chr5H-562950502	5	562920502	C	G	6.42E-05	Ethylene-responsive transcription factor 1
2019 WSL-4	chr1H-22266723	1	22266723	G	A	6.39E-05	alcohol dehydrogenase 1
	chr2H-250021560	2	250021560	T	G	1.39E-05	MATE efflux family protein
	chr2H-258433925	2	258433925	A	G	3.46E-05	Glucan endo-1,3-beta-glucosidase 1
	chr2H-621475962	2	621475962	C	T	1.38E-05	S-adenosylmethionine decarboxylase proenzyme
	chr2H-767327252	2	767327252	C	A	1.21E-05	Peroxidase superfamily protein
	chr3H-386903964	3	386903964	G	A	1.35E-05	Chaperone protein DnaJ
	chr3H-570919222	3	570919222	A	T	4.28E-05	External alternative NAD(P)H-ubiquinone oxidoreductase B3, mitochondrial
	chr4H-97729513	4	97729513	G	A	2.89E-05	Abscisic stress-ripening protein 1
	chr6H-26353758	6	26353758	G	C	1.50E-05	ATP-dependent protease La (LON) domain protein
	chr7H-461397916	7	461397916	G	T	4.14E-06	Protein kinase superfamily protein
	chr7H-478156220	7	478156220	G	A	6.20E-05	Peroxidase superfamily protein
2020 WSL-1	chr2H-250021530	2	250021530	C	T	3.31E-05	MATE efflux family protein
	chr2H-258433925	2	258433925	A	G	2.80E-05	Glucan endo-1,3-beta-glucosidase 1
	chr2H-621475919	2	621475919	G	A	5.64E-05	S-adenosylmethionine decarboxylase proenzyme
	chr7H-478156201	7	478156201	G	A	0.000100413	Peroxidase superfamily protein
	chr7H-478156300	7	478156300	A	G	4.45E-05	Peroxidase superfamily protein
2020 WSL-2	chr2H-621250575	2	621250575	C	A	6.42E-05	S-adenosylmethionine decarboxylase proenzyme
	chr3H-388566821	3	388566821	A	G	8.01E-05	Chaperone protein DnaJ
	chr4H-97729513	4	97729513	G	A	1.93E-05	Abscisic stress-ripening protein 1
	chr6H-26353758	6	26353758	G	C	6.27E-05	ATP-dependent protease La (LON) domain protein
	chr7H-478156203	7	478156203	C	A	8.89E-05	Peroxidase superfamily protein
2020 WSL-3	chr1H-22266723	1	22266723	G	A	5.95E-05	alcohol dehydrogenase 1
	chr2H-767327252	2	767327252	C	A	5.30E-06	Peroxidase superfamily protein
	chr3H-570919222	3	570919222	A	T	2.17E-05	External alternative NAD(P)H-ubiquinone oxidoreductase B3, mitochondrial
	chr5H-562950502	5	562920502	A	G	7.81E-05	Ethylene-responsive transcription factor 1
	chr6H-26353758	6	26353758	G	C	6.99E-07	ATP-dependent protease La (LON) domain protein
	chr7H-461397916	7	461397916	G	T	5.96E-05	Protein kinase superfamily protein
2020 WSL-4	chr3H-649787421	3	649787421	C	T	7.30E-05	MADS-box transcription factor family protein
	chr4H-138201763	4	138201763	A	C	1.59E-08	Nuclear pore complex protein Nup98-Nup96
	chr7H-51003579	7	51003579	G	A	1.48E-05	Vesicle-associated membrane protein 713

### RNA-seq analysis of root transcripts in response to waterlogging stress

Several QTLs for waterlogging tolerance have been mapped by the DH population of TX9425 × Franklin (Li et al., 2008). In the present study, the two varieties also showed significant differences in waterlogging tolerance (**Table S2**). To facilitate the comparison, the roots of TX9425 and Franklin were harvested 72 h after waterlogging treatments. We subsequently performed high-throughput RNA-seq using Illumina HiSeq 2500 and obtained an average of 4.86 million reads from each sample. After removing low-mass, joint, and potentially contaminated data, 2.87–7.58 GB data were obtained from each sample, and the Q30 value ranged from 89.41% to 92.33%, indicating the high-quality sequencing data in the RNA-seq experiments (**Table S5**).

**Figure 3:**
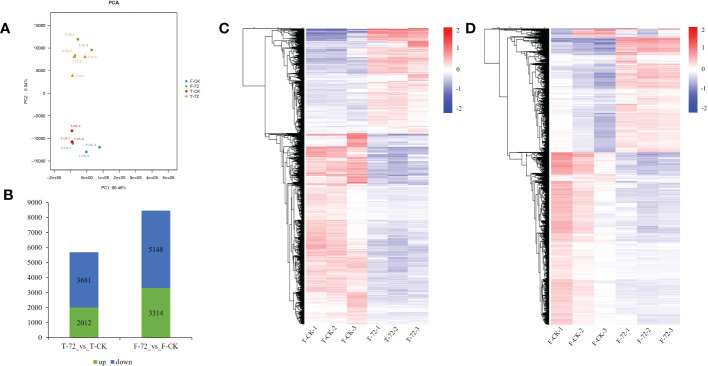
Transcriptome analysis in roots of TX9425 and Franklin under control and waterlogging conditions. **(A)** Principal component analysis (PCA) of transcript changes separates the samples under control and waterlogging (72h waterlogging treatment) conditions. Horizontal and vertical coordinates respectively represent the first and second principal components, and the contribution degree of each principal component is in parentheses. **(B)** Number of upregulated genes (green) and downregulated genes (blue) between barley under waterlogging stress and normal conditions. **(C)** Heatmap clustering of the DEGs in TX9425 according to their expression abundance. **(D)** Heatmap clustering of the DEGs in Franklin according to their expression abundance. The different colors indicate different levels of expression abundance.

A principal component analysis (PCA) was conducted based on the transcriptional profiles. The control and treatment samples of two genotypes could be clearly separated by the first principal component (PC1), which accounted for 99.46 % of the total variation (**Figure 3A**). We identified 5,693 and 8,462 differentially expressed genes (DEGs) under waterlogging treatment (72 h) versus control in TX9425 and Franklin, respectively. We noted that 2,012 DEGs were upregulated and 3,681 DEGs were down-regulated in TX9425, while 3,314 DEGs were up-regulated and 5,148 DEGs were down-regulated in Franklin (**Figures 3B–D**). The gene ontology (GO) functional classification analysis was performed to categorize the DEGs. After 72 h of waterlogging, the DEGs in the two genotypes were mainly functional in binding, catalytic activity, antioxidant activity, cellular anatomical entity, response to stimulus, metabolic process, biological regulation, cellular process and localization (**Figure S4**; **Table S6**).

### Responses of transcription factors to waterlogging

**Figure 4:**
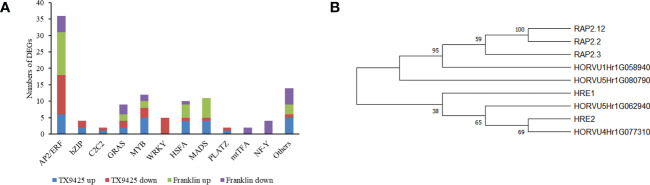
Differentially expressed genes (DEGs) associated with the transcription factor (TF) activity in response to barley waterlogging tolerance. **(A)** Twelve different TF families representing highest number of up- and down-regulated DEGs. **(B)** Phylogenetic tree of barley and *Arabidopsis* ERF VII proteins. Full-length protein sequences were analyzed using Neighbor-joining method in MEGA software. Numbers above branches indicate the bootstrapped value from 1000 replicates.

Under waterlogging stress, 273 DEGs related to TFs were identified. Of these, 168 TFs were up-regulated in TX9425 and Franklin, and 184 TFs were down-regulated in TX9425 and Franklin at 72 h. The AP2/ERF, bZIP, and MYB families represented the highest number of significantly expressed TFs at 72 h of waterlogging (**Figure 4A**).

The AP2/ERF TFs, in particular ERFVII with conserved N-terminal motif [MCGGAII(A/S)], were previously reported to be associated with waterlogging tolerance in different crop plants (Hinz et al., 2010). This motif has been previously reported to play an important role in low oxygen conditions (Gibbs et al., 2011; Licausi et al., 2011). In this study, four ERFVII-type *HvAP2/ERF* genes, including *HORVU4Hr1G077310*, *HORVU5Hr1G080790*, *HORVU1Hr1G058940* and *HORVU5Hr1G062940*, were found to be differentially expressed. Among these four AP2/ERF TFs, the *HORVU4Hr1G077310* and *HORVU5Hr1G080790* were induced at higher levels in TX9425 than in Franklin. Moreover, phylogenetic analysis revealed that *HORVU4Hr1G077310* and *HORVU5Hr1G062940* were more closely related to *Arabidopsis* ERFVII viz., *HRE1* and *HRE2*, whereas *HORVU5Hr1G080790* and *HORVU1Hr1G058940* were more closely related to *RAP2.3*, *RAP2.2*, and *RAP2.12* (**Figure 4B**). Therefore, these results suggested four TFs with important roles in regulating waterlogging tolerance in barley.

### Combined analysis of GWAS and RNA-seq for screening candidate genes of waterlogging stress tolerance

We combined the GWAS and RNA-seq results to further screen waterlogging tolerance candidate genes. After screening with a region of 100 kb near putative SNPs, 166 candidate genes were found for the 72 significant SNPs (**Table S4**). Of the 166 candidate genes in GWAS, 27 exhibited significantly different expression levels under waterlogging stress relative to the control (**Table 4**). Those candidate genes were mapped on 7 chromosomes, 3 on 1H, 7 on 2H, 4 on 3H, 4 on 4H, 3 on 5H, 1 on 6H, and 5 on 7H, respectively. Of the 27 putative DEGs, 11 were up-regulated and 16 were down-regulated. Among them, 10 exhibited significantly different expressions at two or more different time points. 4 candidate genes (*HORVU3Hr1G053060*, *HORVU3Hr1G095240*, *HORVU4Hr1G024430*, and *HORVU5Hr1G080790*) were significantly induced by waterlogging stress in TX9425 and Franklin. These genes encode the following enzymes: chaperone protein DnaJ (Hv DnaJ), MADS-box transcription factor family protein (HvMADS), nuclear pore complex protein (HvNPC), and ethylene-responsive transcription factor 1 (HvERF1). Comparatively, the four candidate genes except *HvNPC* in the waterlogging-tolerant line (TX9425) had higher expression levels than the waterlogging-sensitive line. *HvERF1* in TX9425 exhibited a 39-fold change which was the highest.

**Table 4:** The differential expression of the putative genes detected in both GWAS and transcriptome sequencing.

GeneID	Position	T-CK	T-72	Log_2_ (fc)	*p* value	F-CK	F-72	Log_2_ (fc)	*p* value	Annotation
HORVU1Hr1G010130	chr1: 23059462-23064407	17.74	2.35	-2.91	0.00	13.88	1.32	-3.39	5.62E-05	alcohol dehydrogenase 1
HORVU1Hr1G010230	chr1: 23219158-23220138	14.60	6.14	-1.25	0.28	13.27	1.45	-3.19	0.00040075	Defensin-like protein
HORVU1Hr1G082250	chr1: 528988587-528990782	30.92	782.58	4.66	0.00	40.65	459.58	3.50	2.05E-05	alcohol dehydrogenase 1
HORVU2Hr1G046410	chr2: 249270844-249271705	16.16	2.85	-2.50	0.00	5.90	2.39	-1.31	0.357144795	MATE efflux family protein
HORVU2Hr1G046530	chr2: 249939061-249942337	58.23	28.25	-1.04	0.34	39.98	7.95	-2.33	0.007513532	Ribonuclease UK114
HORVU2Hr1G047430	chr2: 258846019-258881832	5.52	1.58	-1.80	0.05	3.66	0.15	-4.56	4.91E-07	Glucan endo-1,3-beta-glucosidase 1
HORVU2Hr1G086140	chr2: 621114389-621117212	1013.24	817.64	-0.31	0.88	696.05	632.72	-0.14	0.615583461	S-adenosylmethionine decarboxylase proenzyme
HORVU2Hr1G107350	chr2: 711653899-711656553	2.44	0.31	-2.95	0.00	1.74	0.08	-4.42	1.71E-05	Peroxidase superfamily protein
HORVU2Hr1G127480	chr2: 767283074-767284606	9.11	3.27	-1.48	0.15	0.73	0.46	-0.68	0.202301583	Peroxidase superfamily protein
HORVU2Hr1G127650	chr2: 767602920-767603154	85.45	142.69	0.74	0.12	110.84	37.03	-1.58	0.068568152	Peroxidase superfamily protein
HORVU3Hr1G050320	chr3: 359507412-359509968	11.08	8.42	-0.40	0.89	7.26	1.04	-2.81	0.000800056	Cell growth-regulating nucleolar protein
HORVU3Hr1G053060	chr3: 389274477-389280164	1.98	16.70	3.08	0.00	1.12	7.41	2.72	1.29E-05	Chaperone protein DnaJ
HORVU3Hr1G095240	chr3: 649184868-649185995	2.34	78.13	5.06	0.00	9.18	107.78	3.55	4.25E-08	MADS-box transcription factor family protein
HORVU3Hr1G097160	chr3: 655237978-655243219	25.44	9.39	-1.44	0.14	51.92	4.72	-3.46	0.000174693	Alcohol dehydrogenase
HORVU4Hr1G020030	chr4: 97456114-97457293	484.90	46.85	-3.37	0.00	234.69	13.75	-4.09	4.49E-07	Abscisic stress-ripening protein 1
HORVU4Hr1G024430	chr4: 138776287-138780671	12.69	48.70	1.94	0.00	8.53	63.29	2.89	4.30E-06	Nuclear pore complex protein Nup98-Nup96
HORVU4Hr1G024460	chr4: 139378618-139382267	7.91	18.35	1.21	0.01	5.58	15.73	1.50	0.002731588	alpha/beta-Hydrolases superfamily protein
HORVU4Hr1G080250	chr4: 614656390-614657915	32.85	0.98	-5.06	0.00	8.64	0.20	-5.42	7.87E-09	RING/U-box superfamily protein
HORVU5Hr1G045650	chr5: 353125421-353127300	8.01	46.81	2.55	0.00	8.13	63.35	2.96	6.78E-07	NAC domain protein,
HORVU5Hr1G111870	chr5: 637431028-637433690	165.84	282.78	0.77	0.07	68.96	219.81	1.67	0.001678263	DCD (Development and Cell Death) domain protein
HORVU5Hr1G080790	chr5: 562157978-562159066	3.32	129.73	4.97	0.00	8.80	104.05	3.88	1.08E-08	Ethylene-responsive transcription factor 1
HORVU6Hr1G013240	chr6: 26802224-26804260	17.92	334.62	4.22	0.00	31.54	367.07	3.54	5.96E-08	ATP-dependent protease La (LON) domain protein
HORVU7Hr1G008830	chr7: 11491985-11493095	7.61	0.44	-4.11	0.00	2.31	0.06	-5.26	0.000112765	Glutathione S-transferase family protein
HORVU7Hr1G012940	chr7: 18711043-18716636	9.38	25.18	1.42	0.01	4.90	26.24	2.42	4.88E-05	Peptidyl-prolyl cis-trans isomerase-like 3
HORVU7Hr1G028250	chr7: 51040903-51043976	74.72	176.44	1.24	0.01	57.08	110.04	0.95	0.043933689	vesicle-associated membrane protein 713
HORVU7Hr1G080580	chr7: 478251074-478251490	50.05	21.27	-1.23	0.20	26.63	5.84	-2.19	0.008090979	Cryptochrome DASH
HORVU7Hr1G107210	chr7: 622362695-622364651	20.17	4.65	-2.12	0.01	9.33	2.02	-2.21	0.011240794	UDP-Glycosyltransferase superfamily protein

**Figure 5:**
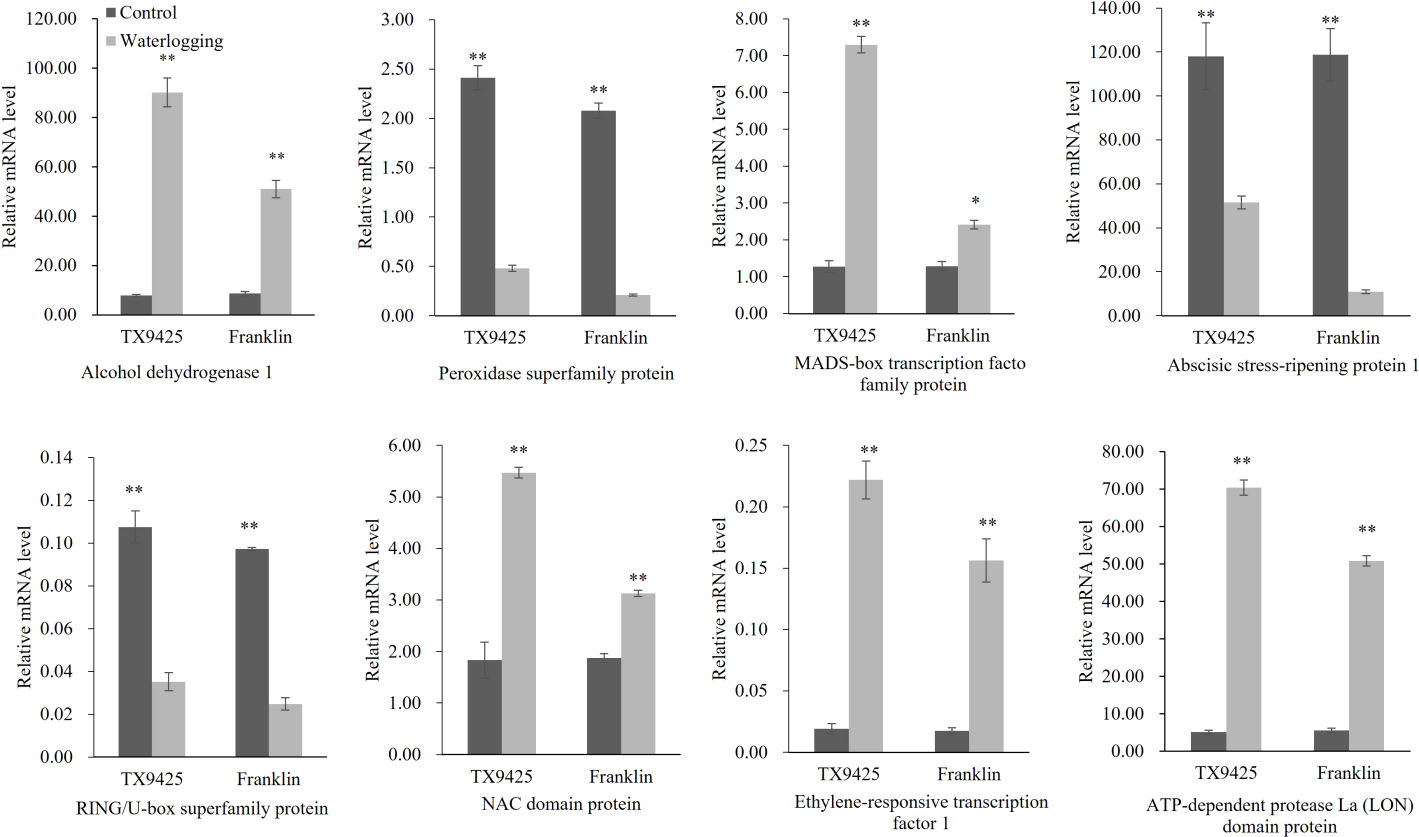
qRT-PCR analysis of eight candidate genes associated with the waterlogging tolerance in barley. “*” means a significant difference at the *P < 0.05* level, “**” means a significant difference at the *P < 0.01* level.

To validate the transcriptional profiles revealed by RNA-seq, qRT-PCR analysis was performed for the eight candidate genes (**Figure 5**). The results showed that the RNA-seq results and qRT-PCR results were highly consistent. Therefore, we speculated that the high expression of these genes is closely related to waterlogging tolerance in barley.

### Overexpression of *HvERF1* in *Arabidopsis* enhances plant waterlogging tolerance

**Figure 6:**
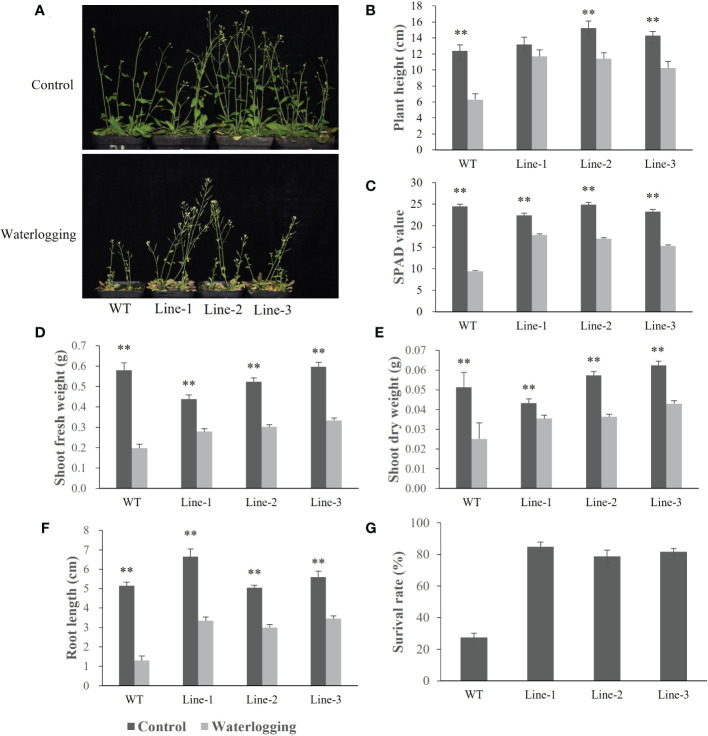
Waterlogging tolerance assay of *HvERF1* overexpression lines (Line1, Line2, Line 3) and wild-type (WT). **(A)** Five week-old plants were subjected to waterlogging stress for further 2 weeks. **(B)** Plant height. **(C)** Soil-plant analysis development (SPAD) value (based on chlorophyll meter reading). **(D)** Shoot fresh weight. **(E)** Shoot dry weight. **(F)** Root length. **(G)** Surival rate in the wild-type and *HvERF1* transgenic lines were measured under control and waterlogging stress. Values are the means ± SD. Means were generated from three independent measurements. Data were analyzed by one-way analysis of variance followed by Duncan’s test. “**” means a significant difference at the *P < 0.01* level.

To investigate the function of barley *HvERF1* (*HORVU5Hr1G080790*), transgenic *Arabidopsis* plants overexpressing the *HvERF1* gene from TX9425 were generated. Five-week-old plants of the wild type (WT) and three homozygous T3 transgenic lines were selected for waterlogging stress experiments. As shown in **Figure 6**, no discernible changes in morphological and developmental phenotypes appeared between the WT and transgenic lines under normal conditions, while the transgenic lines grew better than WT plants after two weeks of waterlogging (**Figure 6A**). Under waterlogging conditions, the plant height was reduced by 49.1% in the WT, and by 11.7%, 11.4%, and 10.3% in the transgenic lines (**Figure 6B**). Compared with the control, the soil and plant analyzer development (SPAD) value was lower 61.6% in the WT, and 20.5%, 31.8%, 34.2% lower in the transgenic lines (**Figure 6C**). The shoot fresh weights of the transgenic lines were 36.1%, 42.3%, and 44.0%, respectively, which were lower than those of the control, while they were 65.8% lower than that in the WT (**Figure 6D**). The shoot dry weight decreased by 51.0% in the WT, and by 18.0%, 36.5% and 31.1% in the transgenic lines (**Figure 6E**). In addition, the root lengths of the WT plants were further reduced compared to those of the transgenic lines during waterlogging stress (**Figure 6F**). Furthermore, the average survival rate of the transgenic lines after waterlogging was 81.8%, but that of the WT was only 27.6% (**Figure 6G**). Altogether, these data indicated that the overexpression of *HvERF1* in *Arabidopsis* significantly enhanced waterlogging tolerance.

### Overexpression of *HvERF1* induced changes in stress-related gene expression levels

**Figure 7:**
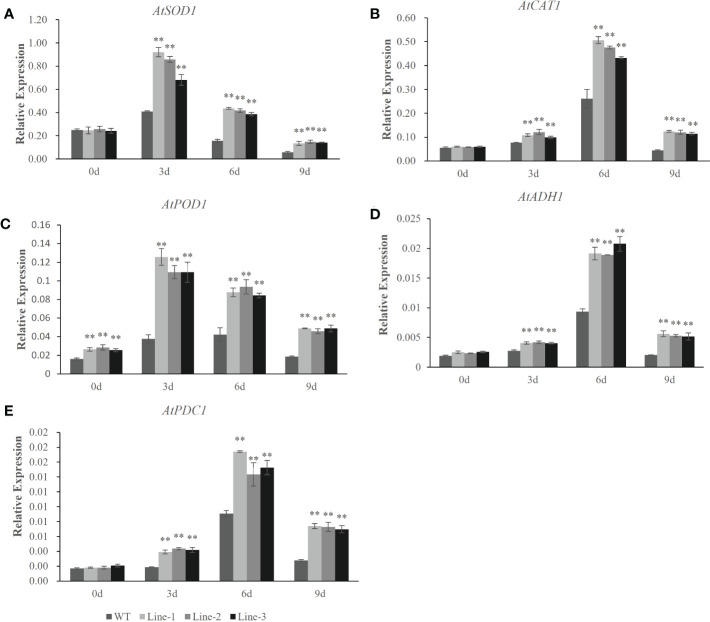
Expression analysis of stress-responsive genes in HvERF1 overexpression lines and WT under waterlogging stresses. The relative expression levels of stress-responsive genes (AtSOD1, AtCAT1, AtPOD1, AtADH1, AtPDC1) were determined by qRT-PCR **(A–E)**. After 3 days, 6 days, 9 days waterlogging treatments, respectively. Seedlings harvested before treatment were used as control. Relative expression levels of these five genes were normalized to the transcripts of AtActin in the same samples. The mean value and standard deviation were obtained from three independent experiments. The data represent mean ± SD of three biological repeats with three measurements per sample. Asterisks indicate significant differences between transgenic plants and WT according to Student’s t-test (** p < 0.01).

To understand the molecular mechanisms of the *HvERF1* responding to waterlogging stress, the transcriptional profiles of five genes related to ROS scavenging and glycolysis (*AtSOD1*, *AtCAT1*, *AtPOD1*, *AtADH1* and *AtPDC1*) were analyzed by qRT-PCR in *HvERF1*-transgenic and WT plants (**Figure 7**). The expression of the stress-related genes, except *AtPOD1*, was not significantly different between the *HvERF1*-transgenic and WT plants under normal conditions. Compared with the control, the expression levels of the five genes were all increased in both the transgenic lines and WT under waterlogging stress, and the increase in the expression level was significantly greater in transgenic lines than in the WT. The expression of *AtSOD1* and *AtPOD1* increased rapidly after waterlogging, reaching peak levels at day 3 of treatment and then decreasing progressively after days 6 and 9 of treatment (**Figure 7A, C**). However, the expression of *AtCAT1*, *AtADH1* and *AtPDC1* increased slowly, reaching maximum levels at day 6, and then decreasing at day 9 (**Figure 7B, D, E**). These results suggested that overexpression of *HvERF1* in *Arabidopsis* might be able to regulate the expression of genes related to antioxidants and fermentation under waterlogging stress conditions.

## Discussion

### The evaluation of waterlogging tolerance in barley

To accurately identify marker-trait associations and QTL, precise phenotyping is essential owning to the complexity of waterlogging tolerance (Zhou, 2011). Different traits have been used to detect the QTL for waterlogging tolerance in barley, such as leaf scoring system, aerenchyma formation, major agronomical traits, carotenoid content, chlorophyll content, and potential membrane maintenance (Zhou, 2011; Zhou et al., 2012; Broughton et al., 2015; Zhang et al., 2016; Zhang et al., 2017; Gill et al., 2017; Gill et al., 2019; Borrego et al., 2021). The WLS and aerenchyma formation have been demonstrated to be the most reliable screening method in barley (Zhou, 2011). However, the measurement of root aerenchyma is labor-intensive and time-consuming, and it cannot be used for high-throughput screening.

In this study, a cement pool experiment and WLS were used to screen and identify waterlogging tolerance in barley. The pool experiment is closer to actual field conditions, and the condition can be better controlled than the pot experiment (Zhou, 2011). The current results revealed a significant variation among barley genotypes under waterlogging treatment. These results suggest that the population was appropriate for use in a GWAS analysis involving barley waterlogging tolerance. Waterlogging stress led to leaf chlorosis, which has been reported in previous studies (Li et al., 2008). Some of the barley genotypes have been reported in response to waterlogging stress. For example, TX9425 from China displayed tolerance to waterlogging stress, while the cultivars Franklin (Australia) and Naso Nijo (Japan) were susceptible (Luan et al., 2018b). In this study, some landraces from the Yangtze River Basin of China were identified with higher waterlogging tolerance, including Liuhesileng and Linanliuleng, among others. These germplasm resources have not been reported before and might represent novel gene sources for waterlogging tolerance in barley.

### Significant SNPs detected with GWAS and previously reported regions

Different marker types and mapping populations have been used to investigate QTLs associated with barley waterlogging tolerance in previous studies (Broughton et al., 2015; Zhang et al., 2016; Gill et al., 2017; Gill et al., 2019; Zhang et al., 2017). A meta-analysis of abiotic stress tolerance QTLs in barley was also reported. Forty-eight QTLs related to waterlogging were identified on all seven chromosomes, and most QTLs were located on chromosomes 2 H and 4 H (Zhang et al., 2016). In this study, the significant SNPs related to barley waterlogging tolerance were mainly concentrated on 2 H (18) and 7 H (15) (**Table S3**). Studies on barley waterlogging tolerance based on GWAS remain relatively scarce. In a study, 247 worldwide spring barley with 35,926 SNPs were used to perform GWAS analysis of barley waterlogging tolerance, and 51 significant associated markers were identified with agronomic and physiological traits. Six novel QTLs and eight candidate genes associated with waterlogging were detected (Borrego et al., 2021).

In this study, GWAS was conducted in 250 barley accessions using 106,131 SNP markers and WLS in different periods of waterlogging treatment. Seventy-two significantly associated markers were detected, and 34 SNPs were detected in at least two environments. The results revealed a complex genetic mechanism of waterlogging tolerance in barley, controlled by multiple small-effect genes. The direct comparison of our GWAS findings with other studies is difficult, as the differences in populations, reference genomes, waterlogging tolerance assessment traits, marker types, and marker densities were used in different studies.

Some associated SNPs in this study overlapped with a number of previously reported regions (**Table S7**). Eleven waterlogging-related QTLs detected in our study are close to the previously reported. The genomic regions (78Mb on 1H, 704 Mb on 2H, 563 Mb on 5H) were major hotspot regions, which were detected multiple times in different populations. The marker chr1H-78215494 was also associated with QHLRL.1H, QHSDW.1H, QHRDW.1H, QHRFW.1H in the Franklin/YYXT mapping population (Broughton et al., 2015), and JHI-Hv50k-2016-19217 used in GWAS (Borrego et al., 2021). The marker chr2H-704331873 was also closely positioned near the QTL GSw1.1, GSw2.1 in Franklin/Yerong, tfsur-1 in Franklin/TX9425, JHI-Hv50k-2016-109151 in nature population (Li et al., 2008; Xue et al., 2010; Borrego et al., 2021). The genomic region 563 Mb on 5H was coincident for the JHI-Hv50k-2016-322832 and JHI-Hv50k-2016-322288 in the natural population, and the QTL yfsur-2 in the DH population of Yerong × Franklin (Li et al., 2008; Borrego et al., 2021). However, compared with the previous reports, some important regions associated with waterlogging were not detected in this study, such as 98cM on 4H and 29Mb on 2H (Broughton et al., 2015; Borrego et al., 2021). Borrego et al. (2021) found that only four markers were associated with WLS traits by GWAS, and three markers (0.37 and 567 Mb on 4H, 554 Mb on 6H) of which were no co-location with the results herein. These results may be related to low-density markers and differences in populations.

### Transcription factors ERFs enhance waterlogging tolerance

Many previous studies have proven that the integrated analysis of GWAS and RNA-seq is useful in detecting candidate genes of complex traits (Yuan et al., 2019; Wang et al., 2022; He et al., 2022). For example, eight candidate genes were identified for tolerance to salt stress in Alfalfa by GWAS coupled with transcriptome analysis (He et al., 2022). Eight candidate genes for forage yield in Sorghum were also identified by this method (Wang et al., 2022). In the present study, 27 DEGs were identified by GWAS and transcriptome sequencing, of which four were significantly up-regulated under waterlogging stress and were detected in different stages. The expression fold changes in *HvDnaJ*, *HvMADS*, and *HvERF1* in TX9425 were more than that in Franklin (**Table 4**). DnaJ (also called HSP40 or J-protein) has been demonstrated in the regulation of physiological pathways, including hormone regulation and plant disease resistance (Liu et al., 2022). In plants, the MADS genes play a positive role in abiotic stresses such as salt, drought, cold, and osmotic stress (Chen et al., 2018).

TFs are known to play a vital role in both abiotic and biotic stress responses. A study reported that several TFs, including MYB, AP2/ERF, NAC, WRKY, and bHLH, were induced under waterlogging stress (Borrego et al., 2020). In the present study, the AP2/ERF families represented the highest number of DEGs in the two genotypes. ERFVIIs play an important role in adjusting to low-oxygen stress. Genes related to low-oxygen stress, such as *SNORKEL*, *SUB 1 A*, *HRE1*, *HRE2*, *RAP2.2*, *RAP2.3*, and *RAP2.12* have been cloned in rice, *Arabidopsis* and belong to the ERFVII (Xu et al., 2006; Hinz et al., 2010; Licausi et al., 2010; Gibbs et al., 2011). In agreement with these studies, our study identified four genes, *HORVU4Hr1G077310*, *HORVU5Hr1G080790*, *HORVU1Hr1G058940* and *HORVU5Hr1G062940*, which up-regulated and possessed conserved N-terminal motif of MCGGAII(A/S). Hence, the results revealed an essential role of AP2/ERF in waterlogging tolerance of barley.

*HvERF1* (*HORVU5Hr1G080790*) in TX9425 showed a 39-fold change by RNA-seq. Intriguingly, *HvERF1* (563 Mb, 5H) was positioned relatively close to the QTL yfsur-2 in DH population of Yerong × Franklin and the JHI-Hv50k-2016-322832, JHI-Hv50k-2016-322288 in the natural population (**Table S7**) (Li et al., 2008; Borrego et al., 2021). Cluster analysis also found that *HvERF1* was closely related to the waterlogging tolerance genes in *Arabidopsis* (*RAP2.3*, *RAP2.2* and *RAP2.12*). Three *Arabidopsis* ERFVII genes improved waterlogging tolerance by directly activating genes related to energy metabolism (Licausi et al., 2010; Licausi et al., 2011). Members of the ERF families have been shown to regulate waterlogging tolerance in wheat, wild soybean and cucumber (Xu et al., 2017; Sharmin et al., 2020; Shen et al., 2020). In this study, overexpression of *HvERF1* in *Arabidopsis* enhanced waterlogging tolerance, protected antioxidant enzyme activities (SOD, POD, and CAT), and increased energy metabolism (ADH). The enhancement of these related indicators may be achieved by *HvERF1* interacting with the downstream specific target genes. Additionally, further experiments are necessary to demonstrate the function of *HvERF1.*

On the whole, this study deployed GWAS and RNA-seq to mine important genes, which might be relevant to waterlogging tolerance in barley, and provided the candidate genes showing waterlogging tolerance applicable in barley molecular breeding.

## Data availability statement

The datasets presented in this study can be found in online repositories. The names of the repository/repositories and accession number(s) can be found in the article/**Supplementary Material**.

## Author contributions

JW conceived and designed the study, supervised the experiments, XX compiled and finalized the article, HYL performed the experiments, CC performed the experiments, JY performed the experiments, HTL analyzed the data, SL analyzed the data, HQ drafted and wrote the manuscript, JZ revised the manuscript, HS revised the manuscript. All authors contributed to the article and approved the submitted version.

## Supplementary material

Supplementary Figure 1 Distribution of 106,131 SNPs on the 7 chromosomes of barley. The horizontal axis shows chromosome length (Mb); the different colors depict SNP density (the number of SNPs per window).

Supplementary Figure 2 Population structure of the 250 accessions. **(A)** Neighbor-joining tree of all 250 barley varieties. **(B)** Principal component analysis of 250 accessions based on genotype. **(C)** Population structure of the 250 accessions based on STRUCTURE when *K* = 2.

Supplementary Figure 3 Manhattan plots resulting from the SNP-based GWAS in waterlogging treatment under different periods. Manhattan plots for 2019 WLS-1, 2019 WLS-2, 2019 WLS-3, 2019 WLS-4, 2020 WLS-1, 2020 WLS-2, 2020 WLS-3, 2020 WLS-4 were shown in **(A-F)**, respectively. The x-axis shows SNP loci along the seven barley chromosomes. The horizontal red line shows the genome-wide significance threshold P-value of –log10 (P-value) value of 4.0. GWAS was performed using the GLM **(Q + K)** model.

Supplementary Figure 4 GO functional enrichment analysis of DEGs in the roots of two barley varieties under the waterlogging and control treatments. **(A)** TX9425 at 72h after waterlogging refer to Control; **(B)** Franklin at 72h after waterlogging refer to Control.
