# Supplementary material for: Genome-wide association scan and transcriptome analysis reveal candidate genes for waterlogging tolerance in cultivated barley
Source: Front Plant Sci. 2022 Dec 15;13:1048939. doi: 10.3389/fpls.2022.1048939 (PMC9798782; doi:10.3389/fpls.2022.1048939)
Supplement: Supplementary Figure 1 — Distribution of 106,131 SNPs on the 7 chromosomes of barley. The horizontal axis shows chromosome length (Mb); the different colors depict SNP density (the number of SNPs per window). [file DataSheet_1.docx]

Table S1 Barley accessions for GWAS analysis

| Accession Number | Barley Germplasm Name | Country of Origin | Row Type | Hulled/Naked |
| --- | --- | --- | --- | --- |
|  |  |  |  |  |
| 1 | Bass | Germany | 2 | Hulled |
| 2 | CDC copdand | Germany | 2 | Hulled |
| 3 | commander | Australian | 2 | Hulled |
| 4 | CONLON | America | 2 | Hulled |
| 5 | Flinder | Germany | 2 | Hulled |
| 6 | Hindmarsh | Australian | 2 | Hulled |
| 7 | Irina | Germany | 2 | Hulled |
| 8 | Metcalfe | Germany | 2 | Hulled |
| 9 | Nendale | Germany | 2 | Hulled |
| 10 | Scope | Germany | 2 | Hulled |
| 11 | Sebastian | Germany | 2 | Hulled |
| 12 | SuYin24 | Japan | 2 | Hulled |
| 13 | WDM01058 | China | 2 | Hulled |
| 14 | ZDM2660 | China | 2 | Hulled |
| 15 | ZDM2678 | China | 2 | Hulled |
| 16 | ZDM84 | China | 6 | Hulled |
| 17 | Morex | America | 6 | Hulled |
| 18 | Bowman | America | 2 | Hulled |
| 19 | CM72 | America | 6 | Hulled |
| 20 | Franklin | Australian | 2 | Hulled |
| 21 | NaSo | Japan | 2 | Hulled |
| 22 | TX9425 | China | 2 | Hulled |
| 23 | Beyewzon | Australian | 2 | Hulled |
| 24 | Aoxuan3 | Australian | 2 | Hulled |
| 25 | Baudin | Australian | 2 | Hulled |
| 26 | Dongjing2 | China | 2 | Hulled |
| 27 | Edamai6 | China | 2 | Hulled |
| 28 | Edamai934 | China | 2 | Hulled |
| 29 | Fengnongpi1 | China | 2 | Hulled |
| 30 | Haiyandamai | China | 6 | Hulled |
| 31 | Pudamai4 | China | 2 | Hulled |
| 32 | Hei09-26 | China | 2 | Hulled |
| 33 | Hu0083 | China | 2 | Hulled |
| 34 | Hua11 | China | 2 | Hulled |
| 35 | Jia3 | China | 2 | Hulled |
| 36 | Kenpi7 | China | 2 | Hulled |
| 37 | Zhepi33 | China | 2 | Hulled |
| 38 | Xihaipi44 | China | 2 | Hulled |
| 39 | Yangnongpi8 | China | 2 | Hulled |
| 40 | Lianpi9115 | China | 2 | Hulled |
| 41 | Yangsimai3号 | China | 2 | Hulled |
| 42 | Yangnongpi11 | China | 2 | Hulled |
| 43 | Yangnongpi12 | China | 2 | Hulled |
| 44 | Supi3 | China | 2 | Hulled |
| 45 | Kenpi15 | China | 6 | Hulled |
| 46 | Zhepi4 | China | 2 | Hulled |
| 47 | Kenpi17 | China | 2 | Hulled |
| 48 | Meinianghuagnjin | Japan | 2 | Hulled |
| 49 | Supi5 | China | 2 | Hulled |
| 50 | Heipi2 | China | 2 | Hulled |
| 51 | Zhenongda3 | China | 2 | Hulled |
| 52 | Yangnongp5 | China | 2 | Hulled |
| 53 | Heipi4 | China | 2 | Hulled |
| 54 | Supi4 | China | 2 | Hulled |
| 55 | Kenpi21119 | China | 2 | Hulled |
| 56 | Dan2 | China | 2 | Hulled |
| 57 | Zhepi8 | China | 2 | Hulled |
| 58 | Lianpi9091 | China | 2 | Hulled |
| 59 | Zheda9 | China | 2 | Hulled |
| 60 | Pudamai8 | China | 2 | Hulled |
| 61 | Pudamai9 | China | 2 | Hulled |
| 62 | Mengpi1 | China | 2 | Hulled |
| 63 | Mengpi3 | China | 2 | Hulled |
| 64 | Mengpi4 | China | 6 | Hulled |
| 65 | Zhepi3 | China | 2 | Hulled |
| 66 | Zhepi2 | China | 2 | Hulled |
| 67 | Zhepi6 | China | 2 | Hulled |
| 68 | Baodamai11 | China | 6 | Hulled |
| 69 | Baodamai12 | China | 2 | Hulled |
| 70 | Baodamai13 | China | 6 | Hulled |
| 71 | Baodamai8 | China | 6 | Hulled |
| 72 | Baodamai14 | China | 6 | Hulled |
| 73 | Edamai7 | China | 2 | Hulled |
| 74 | Edamai934 | China | 2 | Hulled |
| 75 | Zhudamai3 | China | 2 | Hulled |
| 76 | Zhudamai4 | China | 6 | Hulled |
| 77 | Zhudamai5 | China | 2 | Hulled |
| 78 | Zhudamai | China | 2 | Hulled |
| 79 | Kenpimai5 | China | 6 | Hulled |
| 80 | Kenpimai6 | China | 2 | Hulled |
| 81 | Kenpimai8 | China | 2 | Hulled |
| 82 | Kenpimai10 | China | 2 | Hulled |
| 83 | Kenpimai11 | China | 2 | Hulled |
| 84 | Fengdamai16 | China | 2 | Hulled |
| 85 | Fengdamai17 | China | 2 | Hulled |
| 86 | Fengdamai7 | China | 2 | Hulled |
| 87 | Fengdamai9 | China | 2 | Hulled |
| 88 | Fengdamai10 | China | 2 | Hulled |
| 89 | Fengdamai11 | China | 2 | Hulled |
| 90 | Fengdamai13 | China | 2 | Hulled |
| 91 | Fengdamai14 | China | 2 | Hulled |
| 92 | Yangnongpi7 | China | 2 | Hulled |
| 93 | Huadamai1 | China | 2 | Hulled |
| 94 | Huadamai2 | China | 2 | Hulled |
| 95 | Huadamai3 | China | 6 | Hulled |
| 96 | Huadamai4 | China | 6 | Hulled |
| 97 | Huadamai5 | China | 2 | Hulled |
| 98 | Huadamai6 | China | 2 | Hulled |
| 99 | Huadamai7 | China | 2 | Hulled |
| 100 | Huadamai8 | China | 2 | Hulled |
| 101 | Huadamai9 | China | 2 | Hulled |
| 102 | Huadamai10 | China | 6 | Hulled |
| 103 | Xinpi1 | China | 2 | Hulled |
| 104 | Xinpi2 | China | 2 | Hulled |
| 105 | Xinpi6 | China | 2 | Hulled |
| 106 | Supu7 | China | 2 | Hulled |
| 107 | Yan05184 | China | 2 | Hulled |
| 108 | Yan99175 | China | 2 | Hulled |
| 109 | Yan08191 | China | 2 | Hulled |
| 110 | Yan03174 | China | 2 | Hulled |
| 111 | Yunpi2 | China | 6 | Hulled |
| 112 | Yunpi10 | China | 2 | Hulled |
| 113 | Yunpi11 | China | 2 | Hulled |
| 114 | Yunpi12 | China | 2 | Hulled |
| 115 | Yunpi14 | China | 2 | Hulled |
| 116 | Xinpi3 | China | 2 | Hulled |
| 117 | Xinpi5 | China | 2 | Hulled |
| 118 | Xinpi9 | China | 2 | Hulled |
| 119 | Zhexiu22 | China | 2 | Hulled |
| 120 | Zhepi9 | China | 2 | Hulled |
| 121 | Zhe05-50 | China | 2 | Hulled |
| 122 | Zhe05-71 | China | 2 | Hulled |
| 123 | Zhenongda7 | China | 2 | Hulled |
| 124 | Russia 57 | Russia | 2 | Hulled |
| 125 | Falcon | France | 2 | Hulled |
| 126 | Sterling | France | 6 | Hulled |
| 127 | Zhe04-147 | China | 2 | Hulled |
| 128 | Zhe04-164 | China | 2 | Hulled |
| 129 | Zhe05-55 | China | 2 | Hulled |
| 130 | Zhe04-9 | China | 2 | Hulled |
| 131 | Zhe09-079 | China | 6 | Hulled |
| 132 | Zhe09-080 | China | 2 | Hulled |
| 133 | Zhe05-071 | China | 2 | Hulled |
| 134 | Zhu200501 | China | 6 | Hulled |
| 135 | Zhe05-074 | China | 2 | Hulled |
| 136 | Zheda09-048 | China | 2 | Hulled |
| 137 | E97434 | China | 2 | Hulled |
| 138 | Zhe10-052 | China | 2 | Hulled |
| 139 | Zhe10-098 | China | 2 | Hulled |
| 140 | Zhe10-192 | China | 2 | Hulled |
| 141 | Zhe02-28 | China | 2 | Hulled |
| 142 | Zhe07-144 | China | 2 | Hulled |
| 143 | Yan91048 | China | 2 | Hulled |
| 144 | Yanxuan1 | China | 2 | Hulled |
| 145 | Zhe98-26 | China | 2 | Hulled |
| 146 | Jipi3 | China | 2 | Hulled |
| 147 | Jipi2 | China | 2 | Hulled |
| 148 | Zhenongda3 | China | 2 | Hulled |
| 149 | Lianpi1 | China | 2 | Hulled |
| 150 | Edamai8 | China | 2 | Hulled |
| 151 | Pudamai7 | China | 2 | Hulled |
| 152 | Russia 57 | Russia | 6 | Hulled |
| 153 | CAN 8854 | Canada | 2 | Hulled |
| 154 | Jiangningqingpi | China | 6 | Hulled |
| 155 | Tongu bori | France | 6 | Hulled |
| 156 | Orge d'Hiver 113 | France | 6 | Hulled |
| 157 | Maodamai | China | 6 | Hulled |
| 158 | Carola | France | 6 | Hulled |
| 159 | Xiaozhanmang | China | 6 | Hulled |
| 160 | Guangqu2 | Japan | 6 | Hulled |
| 161 | Jiamusi2 | China | 6 | Hulled |
| 162 | C.I.16138Algerian | Algerian | 6 | Hulled |
| 163 | Changshudongyangdamai | Japan | 6 | Hulled |
| 164 | HOR3298 | Iran | 2 | Hulled |
| 165 | Misato Golden | Japan | 2 | Hulled |
| 166 | Atlanta | Canada | 6 | Hulled |
| 167 | Bronco Marriott | Germany | 6 | Hulled |
| 168 | Prior | Australia | 6 | Hulled |
| 169 | Liuhesileng | China | 6 | Hulled |
| 170 | Hiberna | Germany | 6 | Hulled |
| 171 | Linanliuleng | China | 6 | Hulled |
| 172 | Perga | Greece | 6 | Hulled |
| 173 | Yanchengzaoliuleng | China | 6 | Hulled |
| 174 | Jorlnson | Canada | 6 | Hulled |
| 175 | Bangxingdamai | China | 6 | Naked |
| 176 | Linshan | China | 2 | Hulled |
| 177 | American standed | American | 6 | Hulled |
| 178 | Liulengdamai | China | 6 | Naked |
| 179 | Argyle | Australia | 6 | Hulled |
| 180 | Helena | Australia | 6 | Hulled |
| 181 | Taihoku A | Japan | 6 | Hulled |
| 182 | Liulengzidamai | China | 6 | Hulled |
| 183 | Bulgarian 347 | Bulgarian | 6 | Hulled |
| 184 | Favorit | Hungary | 6 | Hulled |
| 185 | Changmangdamai | China | 6 | Hulled |
| 186 | Gairdner | Australia | 6 | Hulled |
| 187 | Rugaohongmang | China | 6 | Hulled |
| 188 | Franka | Germany | 6 | Hulled |
| 189 | AcBurman | American | 6 | Hulled |
| 190 | Suyin27 | Japan | 6 | Hulled |
| 191 | Changdamai | China | 6 | Hulled |
| 192 | OR71 | American | 6 | Hulled |
| 193 | Yizhengsilengzi | China | 6 | Hulled |
| 194 | Eqicaomai | China | 6 | Hulled |
| 195 | F10 BEI 324 | Japan | 6 | Hulled |
| 196 | HOR4229 | Turkey | 6 | Hulled |
| 197 | Smooth Aun86 | Germany | 6 | Hulled |
| 198 | Yangao Damai | China | 6 | Hulled |
| 199 | Монпкалъм | Russia | 6 | Hulled |
| 200 | Chiba damai | China | 6 | Hulled |
| 201 | Mang damai | China | 6 | Hulled |
| 202 | Pinzhognhao32121 | Turkey | 6 | Hulled |
| 203 | HOR1015 | Greece | 6 | Hulled |
| 204 | Taixingzisileng | China | 6 | Naked |
| 205 | Teisen | Korea | 6 | Hulled |
| 206 | HOR996 | Greece | 6 | Hulled |
| 207 | Fenghualiulengyang damai | China | 6 | Hulled |
| 208 | Hosokawanishiki | Japan | 6 | Hulled |
| 209 | Chifenghei | China | 6 | Hulled |
| 210 | Banjo | France | 6 | Hulled |
| 211 | Opal | Netherlands | 6 | Hulled |
| 212 | Funingbai | China | 6 | Hulled |
| 213 | Badia | Syria | 6 | Hulled |
| 214 | Mokusekko 3 | Japan | 6 | Hulled |
| 215 | No. 1337 | Bulgaria | 6 | Hulled |
| 216 | Kawa Bako | Korea | 6 | Hulled |
| 217 | Tianxing | Japan | 2 | Hulled |
| 218 | Dongjing2 | China | 6 | Hulled |
| 219 | Pinzhonghao 37337 | Turkey | 2 | Hulled |
| 220 | Suwon No. 5 | American | 6 | Hulled |
| 221 | Shengxianzaosileng | China | 6 | Hulled |
| 222 | HOR3298 | Iran | 6 | Hulled |
| 223 | Rudognzaoliuleng | China | 6 | Naked |
| 224 | Wuhu60 | China | 6 | Naked |
| 225 | Siyuehuang | China | 6 | Hulled |
| 226 | Changbaimangpi damai | China | 6 | Hulled |
| 227 | Shijiazhuang damai | China | 2 | Hulled |
| 228 | Gloria"s"/Come"s" | Mexica | 6 | Hulled |
| 229 | HOR1014 | Greece | 6 | Hulled |
| 230 | HOR4162 | Ukraine | 6 | Hulled |
| 231 | Daishanliuleng | China | 6 | Hulled |
| 232 | Xinchangliuleng | China | 6 | Hulled |
| 233 | Liulengduanmanggulaomai | China | 6 | Hulled |
| 234 | Zanqiluo | Japan | 6 | Hulled |
| 235 | Jackson No. 1 | American | 6 | Hulled |
| 236 | Pingliangdongdamai | China | 6 | Hulled |
| 237 | Robust | American | 6 | Hulled |
| 238 | Omugi zairai shu | Korea | 6 | Hulled |
| 239 | Brunhild | unkown | 6 | Hulled |
| 240 | Liulengzidamai | China | 6 | Hulled |
| 241 | Jiangjunmudamai | China | 6 | Naked |
| 242 | Supi7 | China | 2 | Hulled |
| 243 | Supi8 | China | 2 | Hulled |
| 244 | Supi11 | China | 2 | Hulled |
| 245 | Yan05028 | China | 2 | Hulled |
| 246 | Yan04189 | China | 2 | Hulled |
| 247 | Supi6 | China | 2 | Hulled |
| 248 | Yan05170 | China | 2 | Hulled |
| 249 | Yan08218 | China | 2 | Hulled |
| 250 | Yan09085 | China | 2 | Hulled |

Table S3 Significant SNPs identified by GWAS with a GLM

| Traits | Marker Name | Chr | POS | REF | ALT | *p* value |
| --- | --- | --- | --- | --- | --- | --- |
| 2019 WLS-1 | chrUn-51514485 | chrUn | 51514485 | T | C | 5.26E-05 |
|  | chrUn-51514510 | chrUn | 51514510 | G | A | 6.69E-05 |
|  | chrUn-51514533 | chrUn | 51514533 | C | A | 5.26E-05 |
|  | chr1H-179727321 | chr1H | 179727321 | C | G | 9.42E-05 |
|  | chr1H-303506331 | chr1H | 303506331 | G | A | 9.04E-05 |
|  | chr1H-316759290 | chr1H | 316759290 | G | A | 1.61E-05 |
|  | chr1H-316759345 | chr1H | 316759345 | A | T | 1.61E-05 |
|  | chr1H-316759402 | chr1H | 316759402 | C | A | 1.61E-05 |
|  | chr1H-327964580 | chr1H | 327964580 | C | T | 8.65E-05 |
|  | chr1H-430543162 | chr1H | 430543162 | G | T | 1.16E-05 |
|  | chr1H-430989550 | chr1H | 430989550 | G | T | 9.58E-05 |
|  | chr1H-470250740 | chr1H | 470250740 | A | T | 6.96E-05 |
|  | chr1H-470832899 | chr1H | 470832899 | T | C | 9.39E-05 |
|  | chr1H-513262590 | chr1H | 513262590 | G | A | 1.82E-05 |
|  | chr2H-370735701 | chr2H | 370735701 | T | C | 9.12E-05 |
|  | chr2H-555234917 | chr2H | 555234917 | T | A | 4.91E-06 |
|  | chr2H-555234942 | chr2H | 555234942 | T | C | 4.22E-06 |
|  | chr2H-682762936 | chr2H | 682762936 | T | C | 3.64E-05 |
|  | chr3H-580312963 | chr3H | 580312963 | G | C | 4.42E-05 |
|  | chr3H-580312977 | chr3H | 580312977 | A | G | 6.63E-05 |
|  | chr3H-655318138 | chr3H | 655318138 | G | A | 1.85E-05 |
|  | chr4H-39199959 | chr4H | 39199959 | T | G | 7.43E-05 |
|  | chr4H-59044257 | chr4H | 59044257 | C | T | 7.52E-05 |
|  | chr4H-59044294 | chr4H | 59044294 | A | G | 5.66E-05 |
|  | chr4H-234468346 | chr4H | 234468346 | C | A | 8.32E-05 |
|  | chr4H-284519469 | chr4H | 284519469 | T | G | 9.76E-05 |
|  | chr4H-321615179 | chr4H | 321615179 | C | T | 4.28E-05 |
|  | chr4H-595937115 | chr4H | 595937115 | A | G | 5.40E-05 |
|  | chr5H-95423975 | chr5H | 95423975 | A | G | 8.38E-05 |
|  | chr5H-373361515 | chr5H | 373361515 | C | T | 5.07E-05 |
|  | chr5H-639551754 | chr5H | 639551754 | A | G | 8.07E-05 |
|  | chr7H-18787333 | chr7H | 18787333 | G | A | 9.73E-05 |
|  | chr7H-51003579 | chr7H | 51003579 | G | A | 9.71E-05 |
|  | chr7H-364325842 | chr7H | 364325842 | G | A | 7.86E-05 |
|  | chr7H-453544107 | chr7H | 453544107 | G | A | 2.24E-05 |
|  | chr7H-453544118 | chr7H | 453544118 | C | T | 6.53E-06 |
|  | chr7H-538938378 | chr7H | 538938378 | G | C | 6.56E-05 |
|  | chr7H-631206397 | chr7H | 631206397 | C | G | 7.06E-05 |
| 2019 WSL-2 | chr1H-14635831 | chr1H | 14635831 | C | T | 6.14E-05 |
|  | chr1H-78215494 | chr1H | 78215494 | G | A | 4.17E-05 |
|  | chr1H-105985672 | chr1H | 105985672 | C | G | 8.37E-05 |
|  | chr1H-317669340 | chr1H | 317669340 | A | G | 9.90E-05 |
|  | chr1H-447509997 | chr1H | 447509997 | T | C | 3.33E-05 |
|  | chr2H-69428461 | chr2H | 69428461 | C | T | 8.25E-05 |
|  | chr2H-249486609 | chr2H | 249486609 | C | G | 3.49E-05 |
|  | chr2H-249486624 | chr2H | 249486624 | T | A | 3.49E-05 |
|  | chr3H-655782956 | chr3H | 655782956 | T | A | 5.67E-05 |
|  | chr3H-658112054 | chr3H | 658112054 | G | A | 3.25E-05 |
|  | chr3H-658112063 | chr3H | 658112063 | G | A | 3.88E-05 |
|  | chr3H-658916704 | chr3H | 658916704 | C | T | 1.00E-04 |
|  | chr3H-658919569 | chr3H | 658919569 | G | A | 7.92E-05 |
|  | chr4H-54300727 | chr4H | 54300727 | C | T | 4.25E-05 |
|  | chr4H-138201763 | chr4H | 138201763 | A | C | 7.03E-05 |
|  | chr4H-562007911 | chr4H | 562007911 | G | A | 7.09E-05 |
|  | chr4H-614692824 | chr4H | 614692824 | G | A | 3.41E-05 |
|  | chr4H-614692859 | chr4H | 614692859 | G | A | 1.93E-05 |
|  | chr7H-18787345 | chr7H | 18787345 | A | G | 2.17E-05 |
| 2019 WSL-3 | chr1H-352577846 | chr1H | 352577846 | C | G | 4.767E-05 |
|  | chr1H-470600925 | chr1H | 470600925 | C | T | 8.55E-05 |
|  | chr2H-4639399 | chr2H | 4639399 | G | A | 8.09E-09 |
|  | chr2H-79159550 | chr2H | 79159550 | C | T | 5.99E-05 |
|  | chr2H-461208215 | chr2H | 461208215 | G | A | 7.52E-05 |
|  | chr2H-467055961 | chr2H | 467055961 | C | T | 4.21E-05 |
|  | chr2H-662167220 | chr2H | 662167220 | G | A | 3.76E-05 |
|  | chr3H-257363408 | chr3H | 257363408 | A | G | 6.60E-05 |
|  | chr3H-649459901 | chr3H | 649459901 | T | C | 2.91E-05 |
|  | chr3H-649459970 | chr3H | 649459970 | C | G | 1.36E-05 |
|  | chr3H-649787421 | chr3H | 649787421 | C | T | 3.23E-05 |
|  | chr3H-653704399 | chr3H | 653704399 | C | T | 8.82E-05 |
|  | chr3H-699416377 | chr3H | 699416377 | G | A | 3.54E-05 |
|  | chr3H-699578728 | chr3H | 699578728 | G | A | 2.13E-05 |
|  | chr4H-3679449 | chr4H | 3679449 | G | A | 7.37E-05 |
|  | chr4H-138201763 | chr4H | 138201763 | A | C | 1.06E-06 |
|  | chr4H-139673815 | chr4H | 139673815 | G | A | 2.55E-05 |
|  | chr4H-154207356 | chr4H | 154207356 | G | A | 6.69E-05 |
|  | chr4H-231596530 | chr4H | 231596530 | G | A | 3.41E-05 |
|  | chr4H-313772418 | chr4H | 313772418 | T | A | 8.26E-05 |
|  | chr4H-396638744 | chr4H | 396638744 | C | T | 4.56E-05 |
|  | chr5H-32913321 | chr5H | 32913321 | A | C | 9.12E-05 |
|  | chr5H-127605370 | chr5H | 127605370 | C | A | 2.37E-05 |
|  | chr5H-136652074 | chr5H | 136652074 | C | T | 8.65E-05 |
|  | chr5H-165036429 | chr5H | 165036429 | C | G | 3.43E-05 |
|  | chr5H-174015099 | chr5H | 174015099 | G | A | 1.08E-05 |
|  | chr5H-193105302 | chr5H | 193105302 | T | C | 8.46E-05 |
|  | chr5H-276482608 | chr5H | 276482608 | T | C | 5.30E-05 |
|  | chr5H-276482628 | chr5H | 276482628 | T | A | 4.38E-05 |
|  | chr5H-280410543 | chr5H | 280410543 | C | T | 4.29E-05 |
|  | chr5H-296303721 | chr5H | 296303721 | T | C | 4.25E-05 |
|  | chr5H-300652140 | chr5H | 300652140 | A | G | 9.07E-05 |
|  | chr5H-300652249 | chr5H | 300652249 | G | T | 9.07E-05 |
|  | chr5H-319964056 | chr5H | 319964056 | A | C | 8.06E-05 |
|  | chr5H-324527225 | chr5H | 324527225 | T | C | 3.70E-05 |
|  | chr5H-327063725 | chr5H | 327063725 | G | C | 4.62E-05 |
|  | chr5H-331281553 | chr5H | 331281553 | T | G | 4.53E-05 |
|  | chr5H-335185276 | chr5H | 335185276 | T | A | 4.90E-06 |
|  | chr5H-516179430 | chr5H | 516179430 | G | A | 1.46E-05 |
|  | chr5H-562950502 | chr5H | 562950502 | C | G | 1.38E-05 |
|  | chr5H-562950536 | chr5H | 562950536 | G | A | 1.38E-05 |
|  | chr5H-626515636 | chr5H | 626515636 | A | G | 5.19E-05 |
|  | chr5H-626515705 | chr5H | 626515705 | C | T | 7.29E-05 |
|  | chr5H-637409954 | chr5H | 637409954 | C | T | 5.54E-06 |
|  | chr6H-582034852 | chr6H | 582034852 | G | A | 4.42E-05 |
|  | chr7H-36283977 | chr7H | 36283977 | G | A | 9.73E-05 |
|  | chr7H-126180900 | chr7H | 126180900 | T | C | 4.37E-05 |
|  | chr7H-512064695 | chr7H | 512064695 | T | C | 8.12E-05 |
|  | chr7H-637225870 | chr7H | 637225870 | C | G | 8.56E-05 |
| 2019 WSL-4 | chr1H-22266723 | chr1H | 22266723 | G | A | 8.28E-05 |
|  | chr1H-23302858 | chr1H | 23302858 | A | G | 2.28E-05 |
|  | chr1H-23302891 | chr1H | 23302891 | G | A | 3.48E-05 |
|  | chr1H-104824510 | chr1H | 104824510 | A | C | 5.23E-05 |
|  | chr1H-104824673 | chr1H | 104824673 | G | A | 5.23E-05 |
|  | chr1H-239052994 | chr1H | 239052994 | A | G | 4.38E-05 |
|  | chr2H-111654194 | chr2H | 111654194 | C | T | 7.11E-05 |
|  | chr2H-250021530 | chr2H | 250021530 | C | T | 1.18E-05 |
|  | chr2H-250021558 | chr2H | 250021558 | C | T | 2.10E-06 |
|  | chr2H-250021560 | chr2H | 250021560 | T | G | 2.10E-06 |
|  | chr2H-258433925 | chr2H | 258433925 | A | G | 3.39E-05 |
|  | chr2H-475198892 | chr2H | 475198892 | G | A | 2.59E-05 |
|  | chr2H-616326637 | chr2H | 616326637 | G | A | 2.27E-05 |
|  | chr2H-616326810 | chr2H | 616326810 | A | C | 5.30E-05 |
|  | chr2H-621250575 | chr2H | 621250575 | C | A | 2.95E-05 |
|  | chr2H-621475919 | chr2H | 621475919 | G | A | 4.38E-06 |
|  | chr2H-621475962 | chr2H | 621475962 | C | T | 4.38E-06 |
|  | chr2H-733231687 | chr2H | 733231687 | T | C | 2.82E-05 |
|  | chr2H-739219243 | chr2H | 739219243 | C | T | 9.71E-05 |
|  | chr2H-739219306 | chr2H | 739219306 | A | T | 1.71E-05 |
|  | chr2H-739219352 | chr2H | 739219352 | G | A | 1.71E-05 |
|  | chr2H-760942846 | chr2H | 760942846 | C | T | 3.79E-05 |
|  | chr2H-760942855 | chr2H | 760942855 | G | C | 4.42E-05 |
|  | chr2H-767327252 | chr2H | 767327252 | C | A | 1.95E-05 |
|  | chr3H-189101476 | chr3H | 189101476 | G | A | 7.16E-05 |
|  | chr3H-288205341 | chr3H | 288205341 | A | G | 4.37E-05 |
|  | chr3H-356479031 | chr3H | 356479031 | C | A | 1.89E-07 |
|  | chr3H-358401521 | chr3H | 358401521 | G | T | 1.72E-07 |
|  | chr3H-361068779 | chr3H | 361068779 | G | A | 5.91E-06 |
|  | chr3H-386903964 | chr3H | 386903964 | G | A | 1.72E-07 |
|  | chr3H-402037225 | chr3H | 402037225 | T | C | 1.74E-05 |
|  | chr3H-417625680 | chr3H | 417625680 | T | G | 1.16E-05 |
|  | chr3H-434343223 | chr3H | 434343223 | G | A | 4.80E-05 |
|  | chr3H-455011675 | chr3H | 455011675 | G | A | 2.60E-05 |
|  | chr3H-479723382 | chr3H | 479723382 | G | C | 3.32E-05 |
|  | chr3H-504691978 | chr3H | 504691978 | G | T | 1.35E-05 |
|  | chr3H-570919222 | chr3H | 570919222 | A | T | 1.47E-05 |
|  | chr4H-97729513 | chr4H | 97729513 | G | A | 4.27E-05 |
|  | chr4H-237428725 | chr4H | 237428725 | C | T | 4.59E-05 |
|  | chr4H-237428780 | chr4H | 237428780 | G | T | 4.59E-05 |
|  | chr4H-403531065 | chr4H | 403531065 | C | T | 6.00E-05 |
|  | chr4H-445620023 | chr4H | 445620023 | G | T | 5.70E-05 |
|  | chr5H-153124274 | chr5H | 153124274 | C | G | 1.09E-05 |
|  | chr5H-153124346 | chr5H | 153124346 | G | A | 1.53E-05 |
|  | chr5H-314772108 | chr5H | 314772108 | G | A | 9.72E-05 |
|  | chr5H-460931806 | chr5H | 460931806 | A | G | 9.43E-05 |
|  | chr5H-499229225 | chr5H | 499229225 | G | A | 3.73E-05 |
|  | chr5H-588302774 | chr5H | 588302774 | G | A | 8.80E-05 |
|  | chr6H-26353758 | chr6H | 26353758 | G | C | 5.15E-06 |
|  | chr6H-57421518 | chr6H | 57421518 | C | A | 2.11E-05 |
|  | chr6H-57421624 | chr6H | 57421624 | A | G | 8.67E-05 |
|  | chr6H-57421644 | chr6H | 57421644 | A | G | 4.41E-06 |
|  | chr6H-58027623 | chr6H | 58027623 | C | G | 7.53E-05 |
|  | chr7H-39793533 | chr7H | 39793533 | G | A | 8.02E-05 |
|  | chr7H-445674925 | chr7H | 445674925 | C | T | 5.85E-05 |
|  | chr7H-461397916 | chr7H | 461397916 | G | T | 5.84E-07 |
|  | chr7H-468869672 | chr7H | 468869672 | T | A | 7.28E-06 |
|  | chr7H-478156220 | chr7H | 478156220 | G | A | 6.76E-05 |
|  | chr7H-574633859 | chr7H | 574633859 | A | G | 3.16E-05 |
|  | chr7H-622349662 | chr7H | 622349662 | G | T | 3.72E-06 |
| 2020 WSL-1 | chr1H-481484195 | chr1H | 481484195 | G | C | 3.36E-05 |
|  | chr1H-492144003 | chr1H | 492144003 | G | A | 6.18E-05 |
|  | chr1H-529042971 | chr1H | 529042971 | T | G | 5.95E-05 |
|  | chr1H-529043032 | chr1H | 529043032 | C | G | 3.87E-06 |
|  | chr1H-529043138 | chr1H | 529043138 | G | A | 9.91E-06 |
|  | chr2H-250021530 | chr2H | 250021530 | C | T | 6.17E-06 |
|  | chr2H-250021558 | chr2H | 250021558 | C | T | 5.68E-06 |
|  | chr2H-250021560 | chr2H | 250021560 | T | G | 5.68E-06 |
|  | chr2H-258433925 | chr2H | 258433925 | A | G | 2.72E-05 |
|  | chr2H-475198892 | chr2H | 475198892 | G | A | 4.18E-05 |
|  | chr2H-621475919 | chr2H | 621475919 | G | A | 4.82E-05 |
|  | chr2H-621475962 | chr2H | 621475962 | C | T | 4.82E-05 |
|  | chr2H-756301133 | chr2H | 756301133 | C | T | 1.98E-05 |
|  | chr3H-288205341 | chr3H | 288205341 | A | G | 8.42E-06 |
|  | chr3H-320660893 | chr3H | 320660893 | T | A | 1.51E-05 |
|  | chr3H-346534188 | chr3H | 346534188 | C | T | 1.07E-05 |
|  | chr3H-356479031 | chr3H | 356479031 | C | A | 4.20E-06 |
|  | chr3H-358401521 | chr3H | 358401521 | G | T | 2.07E-06 |
|  | chr3H-359709979 | chr3H | 359709979 | G | C | 6.29E-05 |
|  | chr3H-359710010 | chr3H | 359710010 | T | A | 6.29E-05 |
|  | chr3H-361068779 | chr3H | 361068779 | G | A | 9.77E-05 |
|  | chr3H-368189970 | chr3H | 368189970 | A | G | 8.20E-05 |
|  | chr3H-380953026 | chr3H | 380953026 | C | T | 1.32E-05 |
|  | chr3H-382995533 | chr3H | 382995533 | T | C | 7.11E-05 |
|  | chr3H-384910726 | chr3H | 384910726 | C | G | 9.23E-05 |
|  | chr3H-386903964 | chr3H | 386903964 | G | A | 2.07E-06 |
|  | chr3H-402037225 | chr3H | 402037225 | T | C | 8.13E-05 |
|  | chr3H-417625680 | chr3H | 417625680 | T | G | 5.06E-05 |
|  | chr3H-434343223 | chr3H | 434343223 | G | A | 1.00E-05 |
|  | chr3H-455011675 | chr3H | 455011675 | G | A | 4.32E-06 |
|  | chr3H-476428777 | chr3H | 476428777 | C | T | 9.77E-05 |
|  | chr3H-479723382 | chr3H | 479723382 | G | C | 3.20E-05 |
|  | chr3H-482357980 | chr3H | 482357980 | G | A | 6.36E-05 |
|  | chr3H-504691978 | chr3H | 504691978 | G | T | 6.47E-05 |
|  | chr4H-89376935 | chr4H | 89376935 | G | T | 2.55E-05 |
|  | chr4H-89376952 | chr4H | 89376952 | A | C | 1.63E-05 |
|  | chr5H-1789884 | chr5H | 1789884 | T | A | 5.77E-05 |
|  | chr5H-1789891 | chr5H | 1789891 | T | C | 7.80E-05 |
|  | chr5H-1789901 | chr5H | 1789901 | G | A | 9.45E-05 |
|  | chr5H-27298091 | chr5H | 27298091 | A | G | 6.75E-06 |
|  | chr5H-59455947 | chr5H | 59455947 | A | C | 9.32E-05 |
|  | chr5H-62622074 | chr5H | 62622074 | G | A | 8.37E-05 |
|  | chr5H-153124274 | chr5H | 153124274 | C | G | 2.97E-05 |
|  | chr5H-180280824 | chr5H | 180280824 | T | C | 8.32E-06 |
|  | chr5H-197176516 | chr5H | 197176516 | T | C | 2.09E-05 |
|  | chr5H-198064631 | chr5H | 198064631 | C | T | 8.08E-05 |
|  | chr5H-277024114 | chr5H | 277024114 | C | T | 1.65E-05 |
|  | chr5H-304306894 | chr5H | 304306894 | G | A | 9.92E-05 |
|  | chr5H-304519396 | chr5H | 304519396 | A | G | 1.43E-05 |
|  | chr5H-314667828 | chr5H | 314667828 | T | C | 7.19E-05 |
|  | chr5H-352751574 | chr5H | 352751574 | G | T | 1.55E-05 |
|  | chr5H-563247307 | chr5H | 563247307 | A | G | 8.16E-05 |
|  | chr5H-563247376 | chr5H | 563247376 | G | T | 5.01E-05 |
|  | chr7H-461397916 | chr7H | 461397916 | G | T | 6.01E-05 |
|  | chr7H-478156201 | chr7H | 478156201 | G | A | 7.89E-05 |
|  | chr7H-478156220 | chr7H | 478156220 | G | A | 1.43E-05 |
|  | chr7H-478156300 | chr7H | 478156300 | A | G | 3.74E-05 |
|  | chr7H-557774750 | chr7H | 557774750 | A | G | 3.91E-05 |
|  | chr7H-557774753 | chr7H | 557774753 | T | A | 3.91E-05 |
|  | chr7H-574128634 | chr7H | 574128634 | C | T | 9.41E-05 |
|  | chr7H-574633859 | chr7H | 574633859 | A | G | 8.42E-06 |
|  | chr7H-622349662 | chr7H | 622349662 | G | T | 7.23E-05 |
| 2020 WSL-2 | chr2H-234432940 | chr2H | 234432940 | A | G | 6.75E-05 |
|  | chr2H-621250575 | chr2H | 621250575 | C | A | 3.67E-05 |
|  | chr2H-621475919 | chr2H | 621475919 | G | A | 8.51E-05 |
|  | chr2H-621475962 | chr2H | 621475962 | C | T | 8.51E-05 |
|  | chr2H-711658502 | chr2H | 711658502 | G | C | 2.99E-05 |
|  | chr3H-356479031 | chr3H | 356479031 | C | A | 7.05E-05 |
|  | chr3H-388566821 | chr3H | 388566821 | A | G | 4.82E-05 |
|  | chr3H-477656623 | chr3H | 477656623 | C | T | 3.42E-05 |
|  | chr3H-479764575 | chr3H | 479764575 | T | C | 8.38E-05 |
|  | chr3H-679527717 | chr3H | 679527717 | G | A | 4.30E-05 |
|  | chr4H-97729513 | chr4H | 97729513 | G | A | 7.71E-06 |
|  | chr4H-237428725 | chr4H | 237428725 | C | T | 7.33E-05 |
|  | chr4H-237428780 | chr4H | 237428780 | G | T | 7.33E-05 |
|  | chr4H-640284643 | chr4H | 640284643 | C | A | 5.80E-05 |
|  | chr5H-28968040 | chr5H | 28968040 | A | G | 4.36E-05 |
|  | chr5H-270932878 | chr5H | 270932878 | A | T | 9.60E-05 |
|  | chr6H-26353758 | chr6H | 26353758 | G | C | 2.70E-05 |
|  | chr6H-57421644 | chr6H | 57421644 | A | G | 8.11E-05 |
|  | chr6H-335109673 | chr6H | 335109673 | G | A | 7.13E-05 |
|  | chr7H-11569360 | chr7H | 11569360 | C | T | 9.95E-07 |
|  | chr7H-195661787 | chr7H | 195661787 | A | T | 9.29E-05 |
|  | chr7H-445674925 | chr7H | 445674925 | C | T | 8.72E-06 |
|  | chr7H-461397916 | chr7H | 461397916 | G | T | 5.84E-05 |
|  | chr7H-478156201 | chr7H | 478156201 | G | A | 9.51E-05 |
|  | chr7H-478156203 | chr7H | 478156203 | C | A | 6.24E-05 |
|  | chr7H-478156220 | chr7H | 478156220 | G | A | 1.60E-05 |
| 2020 WSL-3 | chr1H-22266723 | chr1H | 22266723 | G | A | 9.88E-05 |
|  | chr1H-155055317 | chr1H | 155055317 | G | A | 7.62E-05 |
|  | chr1H-239052843 | chr1H | 239052843 | A | G | 5.15E-05 |
|  | chr1H-239052994 | chr1H | 239052994 | A | G | 8.40E-06 |
|  | chr1H-525353886 | chr1H | 525353886 | A | G | 7.72E-05 |
|  | chr1H-525353930 | chr1H | 525353930 | C | T | 7.72E-05 |
|  | chr2H-70171360 | chr2H | 70171360 | C | A | 6.75E-05 |
|  | chr2H-540088832 | chr2H | 540088832 | A | G | 6.13E-05 |
|  | chr2H-540088835 | chr2H | 540088835 | G | A | 3.78E-05 |
|  | chr2H-704331873 | chr2H | 704331873 | T | C | 1.77E-05 |
|  | chr2H-704331898 | chr2H | 704331898 | G | A | 1.46E-05 |
|  | chr2H-733231687 | chr2H | 733231687 | T | C | 4.76E-05 |
|  | chr2H-767327252 | chr2H | 767327252 | C | A | 1.12E-05 |
|  | chr3H-356479031 | chr3H | 356479031 | C | A | 9.29E-05 |
|  | chr3H-358401521 | chr3H | 358401521 | G | T | 4.37E-05 |
|  | chr3H-361068779 | chr3H | 361068779 | G | A | 9.95E-05 |
|  | chr3H-386903964 | chr3H | 386903964 | G | A | 4.37E-05 |
|  | chr3H-569031376 | chr3H | 569031376 | C | A | 5.41E-05 |
|  | chr3H-570919222 | chr3H | 570919222 | A | T | 3.38E-06 |
|  | chr3H-570919238 | chr3H | 570919238 | T | C | 8.35E-05 |
|  | chr3H-570919241 | chr3H | 570919241 | T | C | 6.67E-05 |
|  | chr3H-570919269 | chr3H | 570919269 | T | C | 6.67E-05 |
|  | chr5H-460808739 | chr5H | 460808739 | A | G | 7.32E-06 |
|  | chr5H-461056651 | chr5H | 461056651 | G | A | 9.82E-05 |
|  | chr5H-464421125 | chr5H | 464421125 | T | A | 2.20E-05 |
|  | chr5H-466254174 | chr5H | 466254174 | A | G | 1.82E-05 |
|  | chr5H-466389716 | chr5H | 466389716 | G | A | 7.80E-05 |
|  | chr5H-466496253 | chr5H | 466496253 | C | A | 7.26E-05 |
|  | chr5H-466496309 | chr5H | 466496309 | C | T | 3.25E-05 |
|  | chr5H-466496316 | chr5H | 466496316 | C | T | 5.50E-05 |
|  | chr5H-471342415 | chr5H | 471342415 | C | T | 2.85E-05 |
|  | chr5H-475263656 | chr5H | 475263656 | C | A | 4.65E-06 |
|  | chr5H-475263704 | chr5H | 475263704 | G | A | 1.09E-05 |
|  | chr6H-26353758 | chr6H | 26353758 | G | C | 6.22E-08 |
|  | chr6H-27179830 | chr6H | 27179830 | A | G | 1.52E-05 |
|  | chr6H-571158254 | chr6H | 571158254 | C | T | 1.83E-05 |
|  | chr7H-45751209 | chr7H | 45751209 | C | A | 3.48E-05 |
|  | chr7H-453544107 | chr7H | 453544107 | G | A | 3.37E-05 |
|  | chr7H-461397916 | chr7H | 461397916 | G | T | 6.11E-05 |
|  | chr7H-468869672 | chr7H | 468869672 | T | A | 3.47E-05 |
|  | chr7H-655451721 | chr7H | 655451721 | C | T | 7.03E-05 |
|  | chr7H-655451779 | chr7H | 655451779 | G | C | 7.39E-05 |
| 2020 WSL-4 | chrUn-4084517 | chrUn | 4084517 | C | T | 2.79E-05 |
|  | chrUn-103733722 | chrUn | 103733722 | A | G | 9.74E-05 |
|  | chr1H-14635831 | chr1H | 14635831 | C | T | 2.71E-05 |
|  | chr1H-42645586 | chr1H | 42645586 | C | T | 2.61E-05 |
|  | chr1H-143702901 | chr1H | 143702901 | C | G | 7.20E-05 |
|  | chr1H-317669340 | chr1H | 317669340 | A | G | 9.82E-05 |
|  | chr1H-513262684 | chr1H | 513262684 | C | A | 6.03E-05 |
|  | chr2H-4639399 | chr2H | 4639399 | G | A | 5.25E-05 |
|  | chr2H-10515441 | chr2H | 10515441 | G | A | 2.46E-05 |
|  | chr2H-24394444 | chr2H | 24394444 | C | T | 8.49E-05 |
|  | chr2H-24394532 | chr2H | 24394532 | C | T | 1.13E-05 |
|  | chr2H-116518008 | chr2H | 116518008 | T | C | 5.30E-05 |
|  | chr2H-227000975 | chr2H | 227000975 | G | A | 9.88E-05 |
|  | chr2H-295280938 | chr2H | 295280938 | T | C | 6.61E-05 |
|  | chr2H-353990374 | chr2H | 353990374 | G | A | 1.70E-05 |
|  | chr2H-365530283 | chr2H | 365530283 | C | T | 8.19E-05 |
|  | chr2H-555234917 | chr2H | 555234917 | T | A | 6.88E-05 |
|  | chr2H-598021574 | chr2H | 598021574 | T | C | 6.99E-05 |
|  | chr2H-621082684 | chr2H | 621082684 | A | G | 9.65E-05 |
|  | chr2H-669633457 | chr2H | 669633457 | C | T | 3.02E-05 |
|  | chr3H-394457354 | chr3H | 394457354 | G | A | 6.25E-05 |
|  | chr3H-454402357 | chr3H | 454402357 | C | T | 7.41E-05 |
|  | chr3H-643195918 | chr3H | 643195918 | G | T | 1.00E-04 |
|  | chr3H-649787421 | chr3H | 649787421 | C | T | 5.90E-05 |
|  | chr3H-655318138 | chr3H | 655318138 | G | A | 8.60E-05 |
|  | chr4H-11777112 | chr4H | 11777112 | G | A | 7.24E-05 |
|  | chr4H-138201763 | chr4H | 138201763 | A | C | 2.13E-08 |
|  | chr4H-138201792 | chr4H | 138201792 | G | A | 3.61E-05 |
|  | chr4H-259460268 | chr4H | 259460268 | A | G | 7.59E-05 |
|  | chr4H-482284263 | chr4H | 482284263 | T | C | 9.60E-05 |
|  | chr4H-511134587 | chr4H | 511134587 | C | G | 6.29E-05 |
|  | chr4H-512702741 | chr4H | 512702741 | C | T | 4.32E-05 |
|  | chr4H-614692859 | chr4H | 614692859 | G | A | 4.47E-05 |
|  | chr5H-86310777 | chr5H | 86310777 | T | C | 6.06E-06 |
|  | chr5H-100818998 | chr5H | 100818998 | A | G | 3.95E-05 |
|  | chr5H-124044610 | chr5H | 124044610 | A | G | 9.70E-05 |
|  | chr5H-152048430 | chr5H | 152048430 | A | G | 3.47E-05 |
|  | chr5H-159213893 | chr5H | 159213893 | T | C | 3.25E-05 |
|  | chr5H-172859308 | chr5H | 172859308 | G | A | 1.98E-05 |
|  | chr5H-242095830 | chr5H | 242095830 | C | T | 3.60E-05 |
|  | chr5H-256071673 | chr5H | 256071673 | T | C | 9.82E-05 |
|  | chr5H-268975705 | chr5H | 268975705 | A | G | 3.57E-05 |
|  | chr5H-273795147 | chr5H | 273795147 | G | A | 5.58E-05 |
|  | chr5H-276482608 | chr5H | 276482608 | T | C | 7.49E-05 |
|  | chr5H-276482628 | chr5H | 276482628 | T | A | 3.33E-05 |
|  | chr5H-296303721 | chr5H | 296303721 | T | C | 9.56E-05 |
|  | chr5H-324527225 | chr5H | 324527225 | T | C | 9.09E-05 |
|  | chr5H-497018591 | chr5H | 497018591 | T | C | 9.16E-05 |
|  | chr6H-34773472 | chr6H | 34773472 | A | T | 9.23E-05 |
|  | chr7H-18787333 | chr7H | 18787333 | G | A | 1.99E-05 |
|  | chr7H-51003579 | chr7H | 51003579 | G | A | 1.04E-05 |
|  | chr7H-186154130 | chr7H | 186154130 | T | C | 9.98E-05 |
|  | chr7H-193956446 | chr7H | 193956446 | C | G | 9.90E-05 |
|  | chr7H-333714687 | chr7H | 333714687 | T | C | 4.94E-05 |
|  | chr7H-424659441 | chr7H | 424659441 | A | C | 5.48E-05 |
|  | chr7H-569179639 | chr7H | 569179639 | C | G | 6.05E-05 |
|  | chr7H-638269756 | chr7H | 638269756 | C | G | 1.28E-06 |
|  | chr7H-638269760 | chr7H | 638269760 | A | C | 3.46E-05 |
|  | chr7H-638462648 | chr7H | 638462648 | C | A | 1.25E-05 |
|  | chr7H-638759505 | chr7H | 638759505 | T | C | 8.82E-05 |

Table S4 Significant SNPs identified by GWAS with a MLM

| Traits | Marker Name | Chr | POS | REF | ALT | *p* value | No.of genes |
| --- | --- | --- | --- | --- | --- | --- | --- |
| 2019 WSL-1 | chr1H-430989550 | 1 | 430989550 | G | T | 9.53E-05 | 1 |
|  | chr2H-370735701 | 2 | 370735701 | T | C | 2.43E-05 | 1 |
|  | chr2H-555234942 | 2 | 555234942 | T | C | 5.77E-05 | 2 |
|  | chr3H-655318138 | 3 | 655318138 | G | A | 9.96E-05 | 8 |
|  | chr7H-51003579 | 7 | 51003579 | G | A | 5.50E-05 | 5 |
| 2019 WSL-2 | chr1H-14635831 | 1 | 14635831 | C | T | 8.68E-05 | 1 |
|  | chr1H-78215494 | 1 | 78215494 | G | A | 3.97E-05 | 1 |
|  | chr2H-249486624 | 2 | 249486624 | T | A | 2.17E-05 | 1 |
|  | chr4H-138201763 | 4 | 138201763 | A | C | 7.17E-05 | 2 |
|  | chr4H-614692859 | 4 | 614692859 | G | A | 9.25E-05 | 3 |
|  | chr7H-18787345 | 7 | 18787345 | A | G | 8.61E-05 | 2 |
| 2019 WSL-3 | chr2H-4639399 | 2 | 4639399 | G | A | 2.14E-06 | 10 |
|  | chr3H-649787421 | 3 | 649787421 | C | T | 3.23E-05 | 5 |
|  | chr4H-138201763 | 4 | 138201763 | A | C | 1.24E-06 |  |
|  | chr5H-562950502 | 5 | 562920502 | C | G | 6.42E-05 | 1 |
|  | chr5H-637409954 | 5 | 637409954 | C | T | 6.49E-05 | 5 |
| 2019 WSL-4 | chr1H-22266723 | 1 | 22266723 | G | A | 6.39E-05 | 4 |
|  | chr1H-23302858 | 1 | 23302858 | A | G | 7.10E-05 | 3 |
|  | chr2H-250021560 | 2 | 250021560 | T | G | 1.39E-05 |  |
|  | chr2H-258433925 | 2 | 258433925 | A | G | 3.46E-05 | 1 |
|  | chr2H-621475962 | 2 | 621475962 | C | T | 1.38E-05 |  |
|  | chr2H-760942846 | 2 | 760942846 | C | T | 7.96E-05 | 8 |
|  | chr2H-767327252 | 2 | 767327252 | C | A | 1.21E-05 | 13 |
|  | chr3H-358401521 | 3 | 358401521 | G | T | 1.35E-05 | 2 |
|  | chr3H-386903964 | 3 | 386903964 | G | A | 1.35E-05 | 4 |
|  | chr3H-570919222 | 3 | 570919222 | A | T | 4.28E-05 | 6 |
|  | chr4H-97729513 | 4 | 97729513 | G | A | 2.89E-05 | 5 |
|  | chr6H-26353758 | 6 | 26353758 | G | C | 1.50E-05 | 2 |
|  | chr6H-57421518 | 6 | 57421518 | C | A | 5.08E-05 | 1 |
|  | chr6H-480743733 | 6 | 480743733 | A | C | 9.53E-05 | 4 |
|  | chr7H-39793533 | 7 | 39793533 | G | A | 0.000100767 | 1 |
|  | chr7H-461397916 | 7 | 461397916 | G | T | 4.14E-06 | 2 |
|  | chr7H-468869672 | 7 | 468869672 | T | A | 2.82E-05 | 1 |
|  | chr7H-478156220 | 7 | 478156220 | G | A | 6.20E-05 |  |
|  | chr7H-622349662 | 7 | 622349662 | G | T | 2.78E-05 | 1 |
| 2020 WSL-1 | chr1H-500629342 | 1 | 500629342 | G | A | 9.48E-05 | 1 |
|  | chr1H-529043032 | 1 | 529043032 | C | G | 2.31E-05 | 2 |
|  | chr2H-250021530 | 2 | 250021530 | C | T | 3.31E-05 | 2 |
|  | chr2H-258433925 | 2 | 258433925 | A | G | 2.80E-05 |  |
|  | chr2H-621475919 | 2 | 621475919 | G | A | 5.64E-05 | 1 |
|  | chr5H-197176516 | 5 | 197176516 | T | C | 3.23E-05 | 0 |
|  | chr5H-277024114 | 5 | 277024114 | C | T | 3.32E-05 | 1 |
|  | chr5H-352751574 | 5 | 352751574 | G | T | 8.26E-05 | 1 |
|  | chr7H-478156201 | 7 | 478156201 | G | A | 0.000100413 |  |
|  | chr7H-478156300 | 7 | 478156300 | A | G | 4.45E-05 |  |
|  | chr7H-574633859 | 7 | 574633859 | A | G | 2.16E-05 | 1 |
| 2020 WSL-2 | chr2H-621250575 | 2 | 621250575 | C | A | 6.42E-05 | 4 |
|  | chr2H-711658502 | 2 | 711658502 | G | C | 5.03E-05 | 4 |
|  | chr3H-388566821 | 3 | 388566821 | A | G | 8.01E-05 | 3 |
|  | chr3H-679527717 | 3 | 679527717 | G | A | 7.42E-05 | 8 |
|  | chr4H-97729513 | 4 | 97729513 | G | A | 1.93E-05 |  |
|  | chr5H-28968040 | 5 | 28968040 | A | G | 8.59E-05 | 7 |
|  | chr6H-26353758 | 6 | 26353758 | G | C | 6.27E-05 |  |
|  | chr6H-335109673 | 6 | 335109673 | G | A | 9.64E-05 | 1 |
|  | chr7H-11569360 | 7 | 11569360 | C | T | 3.91E-06 | 7 |
|  | chr7H-445674925 | 7 | 445674925 | C | T | 1.94E-05 | 1 |
|  | chr7H-478156203 | 7 | 478156203 | C | A | 8.89E-05 | 3 |
| 2020 WSL-3 | chr1H-22266723 | 1 | 22266723 | G | A | 5.95E-05 |  |
|  | chr1H-239052994 | 1 | 239052994 | A | G | 6.87E-05 | 2 |
|  | chr2H-704331873 | 2 | 704331873 | T | C | 9.55E-05 | 6 |
|  | chr2H-704331898 | 2 | 704331898 | G | A | 7.03E-05 |  |
|  | chr2H-767327252 | 2 | 767327252 | C | A | 5.30E-06 |  |
|  | chr3H-570919222 | 3 | 570919222 | A | T | 2.17E-05 |  |
|  | chr5H-475263656 | 5 | 475263656 | C | A | 8.16E-05 | 1 |
|  | chr5H-562950502 | 5 | 562920502 | A | G | 7.81E-05 | 2 |
|  | chr6H-26353758 | 6 | 26353758 | G | C | 6.99E-07 |  |
|  | chr7H-461397916 | 7 | 461397916 | G | T | 5.96E-05 |  |
| 2020 WSL-4 | chr1H-143702901 | 1 | 143702901 | C | G | 9.16E-05 | 1 |
|  | chr2H-344602707 | 2 | 344602707 | A | T | 6.26E-05 | 1 |
|  | chr3H-649787421 | 3 | 649787421 | C | T | 7.30E-05 |  |
|  | chr4H-138201763 | 4 | 138201763 | A | C | 1.59E-08 |  |
|  | chr7H-51003579 | 7 | 51003579 | G | A | 1.48E-05 |  |

| Table S5 The information of transcriptome libraries. | | | | | | | | | |
| --- | --- | --- | --- | --- | --- | --- | --- | --- | --- |
| Sample | Raw Data | | Valid Data | | Valid Ratio(reads) | Q20% | Q30% | Mapped reads | Mapped-rate(%) |
|  | Read | Base | Read | Base |  |  |  |  |  |
| F-CK-1 | 48944768 | 7.34G | 37367994 | 5.49G | 74.80 | 97.35 | 91.66 | 19075598 | 51.05 |
| F-CK-2 | 63104144 | 9.47G | 35895532 | 5.27G | 55.65 | 97.59 | 92.33 | 27142785 | 75.62 |
| F-CK-3 | 58105296 | 8.72G | 40261296 | 5.92G | 67.89 | 97.43 | 91.77 | 33457137 | 83.1 |
| F-72-1 | 36657956 | 5.49G | 19557404 | 2.87G | 52.28 | 96.45 | 89.41 | 11013633 | 56.31 |
| F-72-2 | 39333742 | 5.9G | 29585370 | 4.36G | 73.90 | 97.28 | 91.6 | 15544835 | 52.54 |
| F-72-3 | 71452314 | 10.72G | 51723936 | 7.58G | 70.71 | 96.42 | 89.5 | 30282512 | 58.55 |
| T-CK-1 | 43239924 | 6.49G | 36732304 | 5.39G | 83.05 | 97.23 | 91.22 | 17937718 | 48.83 |
| T-CK-2 | 56033606 | 8.41G | 45155982 | 6.64G | 78.95 | 97.52 | 92.18 | 21239852 | 47.04 |
| T-CK-3 | 36124766 | 5.42G | 28501422 | 4.18G | 77.12 | 97.2 | 91.23 | 13970074 | 49.02 |
| T-72-1 | 36134806 | 5.42G | 30971468 | 4.54G | 83.76 | 96.94 | 90.41 | 15850393 | 51.18 |
| T-72-2 | 36320990 | 5.45G | 21130356 | 3.11G | 57.06 | 97.06 | 90.94 | 11167419 | 52.85 |
| T-72-3 | 38626874 | 5.79G | 20069610 | 2.95G | 50.95 | 96.81 | 90.3 | 12431933 | 61.94 |

Table S7 Comparison of previously identified waterlogging-related QTLs with our study.

|  |  |  |  |  |  |  |  |  |  |
| --- | --- | --- | --- | --- | --- | --- | --- | --- | --- |
| Traits | Marker Name | Chr | Physical position (bp)**^a^** | Genetic position (cM)**^b^** | Involved traits in other studies **^c^** | Population | Genetic position (cM) | QTL name | Reference |
| 2019 WSL-1 | chr2H-555234942 | 2 | 555234942 | 57.72 | Waterlogging tolerance score (4 weeks) | Franklin / Yerong | 64.17 | WL4.1 | Zhou 2011 |
|  |  | 2 | 555234942 | 57.72 | Waterlogging tolerance score (9 weeks) | Franklin / YYXT | 64.17 | QWl.YyFr.2H | Zhou et al. 2012 |
|  |  | 2 | 555234942 | 57.72 | Biomass | natural population |  | JHI-Hv50k-2016-95223 | Borrego-Benjumea et al. 2021 |
| 2019 WSL-2 | chr1H-78215494 | 1 | 78215494 | 46.46 | Hypoxia longest root length | Franklin / YYXT | 36.62 | QHLRL.1H | Broughton et al. 2015 |
|  |  | 1 | 78215494 | 46.46 | Hypoxia shoot dry weight | Franklin / YYXT | 37 | QHSDW.1H | Broughton et al. 2015 |
|  |  | 1 | 78215494 | 46.46 | Hypoxia root dry weight | Franklin / YYXT | 50 | QHRDW.1H | Broughton et al. 2015 |
|  |  | 1 | 78215494 | 46.46 | Hypoxia root fresh weight | Franklin / YYXT | 50 | QHRFW.1H | Broughton et al. 2015 |
|  |  | 1 | 78215494 | 46.46 | Waterlogging score | natural population |  | JHI-Hv50k-2016-19217 | Borrego-Benjumea et al. 2021 |
| 2019 WSL-2 | chr4H-614692859 | 4 | 614692859 | 85.84 | Waterlogging tolerance score (3 weeks) | Franklin / Yerong | 91 | WL3.4 | Zhou 2011 |
|  |  | 4 | 614692859 | 85.84 | Waterlogging score | natural population |  | JHI-Hv50k-2016-256186 | Borrego-Benjumea et al. 2021 |
| 2019 WSL-3/2020 WSL-3 | chr5H-562950502 | 5 | 562920502 | 97.51 | Waterlogging plant survival | Franklin / Yerong | 92.29 | yfsur-2 | Li et al. 2008 |
|  |  | 5 | 562920502 | 97.51 | Spike per plant | natural population |  | JHI-Hv50k-2016-322832 | Borrego-Benjumea et al. 2021 |
|  |  | 5 | 562920502 | 97.51 | Kernel weight per plant | natural population |  | JHI-Hv50k-2016-322288 | Borrego-Benjumea et al. 2021 |
|  |  | 5 | 562920502 | 97.51 | Grain per plant | natural population |  | JHI-Hv50k-2016-322832 | Borrego-Benjumea et al. 2021 |
| 2019 WSL-3 | chr5H-637409954 | 5 | 637409954 | 126.25 | Hypoxia root fresh weight | Franklin / YYXT | 132.9 | QHRFW.5H | Broughton et al. 2015 |
|  |  | 5 | 637409954 | 126.25 | Spike per plant | natural population |  | JHI-Hv50k-2016-336773 | Borrego-Benjumea et al. 2021 |
| 2019 WSL-4/2020 WSL-3 | chr2H-767327252 | 2 | 767327252 | 129.78 | Waterlogging tolerance score (9 weeks) | Franklin / Yerong | 129.1 | QWL.YeFr.2H.2 | Zhou 2011 |
|  |  | 2 | 767327252 | 129.78 | Waterlogging tolerance score (5 weeks) | Franklin / Yerong | 138.2 | WL5.3 | Zhou 2011 |
|  |  | 2 | 767327252 | 129.78 | Kernel weight per plant | natural population |  | JHI-Hv50k-2016-132004 | Borrego-Benjumea et al. 2021 |
| 2019 WSL-4 | chr6H-480743733 | 6 | 480743733 | 65.93 | Grains per plant | natural population |  | JHI-Hv50k-2016-410329 | Borrego-Benjumea et al. 2021 |
| 2019 WSL-4 | chr7H-39793533 | 7 | 39793533 | 29.96 | Plant height | natural population |  | JHI-Hv50k-2016-457688 | Borrego-Benjumea et al. 2021 |
| 2020 WSL-2 | chr7H-11569360 | 7 | 11569360 | 11.54 | Grains per plant | natural population | 11.54 | JHI-Hv50k-2016-449124 | Borrego-Benjumea et al. 2021 |
|  |  | 7 | 11569360 | 11.54 | Kernel weight per plant | natural population |  | JHI-Hv50k-2016-449124 | Borrego-Benjumea et al. 2021 |
| 2020 WSL-3 | chr1H-239052994 | 1 | 239052994 | 47.94 | Hypoxia shoot dry weight | Franklin / YYXT | 37 | QHSDW.1H | Broughton et al. 2015 |
|  |  | 1 | 239052994 | 47.94 | Hypoxia root dry weight | Franklin / YYXT | 50 | QHRDW.1H | Broughton et al. 2015 |
|  |  | 1 | 239052994 | 47.94 | Spike per plant | natural population |  | JHI-Hv50k-2016-21022 | Borrego-Benjumea et al. 2021 |
| 2020 WSL-3 | chr2H-704331873 | 2 | 704331873 | 82.51 | Waterlogging grains per spike 05-06 | Franklin / Yerong | 82.13 | GSw1.1 | Xue et al. 2010 |
|  |  | 2 | 704331873 | 82.51 | Waterlogging grains per spike 06-07 | Franklin / Yerong | 82.13 | GSw2.1 | Xue et al. 2010 |
|  |  | 2 | 704331873 | 82.51 | Waterlogging plant survival | Franklin / TX9425 | 92.04 | tfsur-1 | Li et al. 2008 |
|  |  | 2 | 704331873 | 82.51 | Spike per plant | natural population |  | JHI-Hv50k-2016-109151 | Borrego-Benjumea et al. 2021 |
|  |  |  |  |  |  |  |  |  |  |
|  |  |  |  |  |  |  |  |  |  |
| ^a^ Base pair positions of the marker in the chromosome based on a high-quality reference genome assembly for barley (*Hordeum vulgare* L.) | | | | | | | | |  |
| ^b^ Genetic marker positions (cM) of the marker obtained from the POPSEQ_2017 genome map in BarleyMap (http://floresta.eead.csic.es/barleymap/find/). | | | | | | | | |  |
| ^c^ Studies used for comparison included the waterlogging stress-related QTLs studies published on barley from 2008 up to now. | | | | | | | |  |  |
|  |  |  |  |  |  |  |  |  |  |

Table S8 qRT-PCR and cloning primers used in this study

| Primer name | Forward primer (5'- 3') | Reverse primer (5'- 3') | Description |
| --- | --- | --- | --- |
| HvADH1 | CTCGCTTCACCATCAACGGAA | GGGTTGATCTTTGCGAGGCA | qRT-PCR |
| HvPOD1 | GGACCGGCTGTACAACTTCA | GACGTGACGGTAGTAGCTCG | qRT-PCR |
| HvMADS1 | AGTGAAGGCGGTGCAAGTAA | CGCTATTTCGTTGCGCCAAT | qRT-PCR |
| HvASR1 | GAGTACGAGCGGATCACCAA | CTCGATCTTGTGCTTGTGCG | qRT-PCR |
| HvRSP1 | CCATGGACTTCTTGCCGGG | GAGCCCGAACGACGACATAA | qRT-PCR |
| HvNAC1 | CGTGGAGGACTCGCATAACA | GTAGTCGACGGTGTTGAGCA | qRT-PCR |
| HvERF1 | CTCGATCCATTCCCTGGTGG | AAGGCTTTGGACTGGGAAGG | qRT-PCR |
| HvAPL1 | CCAGCCCAAACAAAGGGCTC | CTTGCTTGCGGTCTTGAGTG | qRT-PCR |
| Hvactin | GGTCCATCCTAGCCTCACTC | GATAACAGCAGTGGAGCGCT | qRT-PCR |
| AtSOD1 | GTTGGTAGGGCTGTTGTTGTC | TGGACCTCCTTATTACATCAA | qRT-PCR |
| AtCAT1 | AGCGCTTTCGGAGCCTCGTG | GGCCTCACGTTAAGACGAGTTGC | qRT-PCR |
| AtPOD1 | GCACATACGATAGGAGTCACACA | GACAAGCAACACGCAAGAAC | qRT-PCR |
| AtADH1 | TTGCTCCACCGCAGAAACAC | CCAACACTCTCAACAATCCCTCC | qRT-PCR |
| AtPDC1 | TGATGCTTCAGGCTATGCTTT | GTTGAAGATTGGACCTGCAAA | qRT-PCR |
| Atactin-F | CCAACAGAGAGAAGATGA | ATGTCTCTTACAATTTCCCG | qRT-PCR |
| HvERF1 | AACACCCTTCCCAGTCCAAA | TGCCCCGATGTGATTTCACA | Gene cloning |
| Gate-25 | GGGGACAAGTTTGTACAAAAAAGCAGGCTTCATGCCGCCCGCAGCCATGGC | GGGGGACCACTTTGTACAAGAAAGCTGGGTCCTACTCGTTTGCGGCGGCGG | Vector construction |


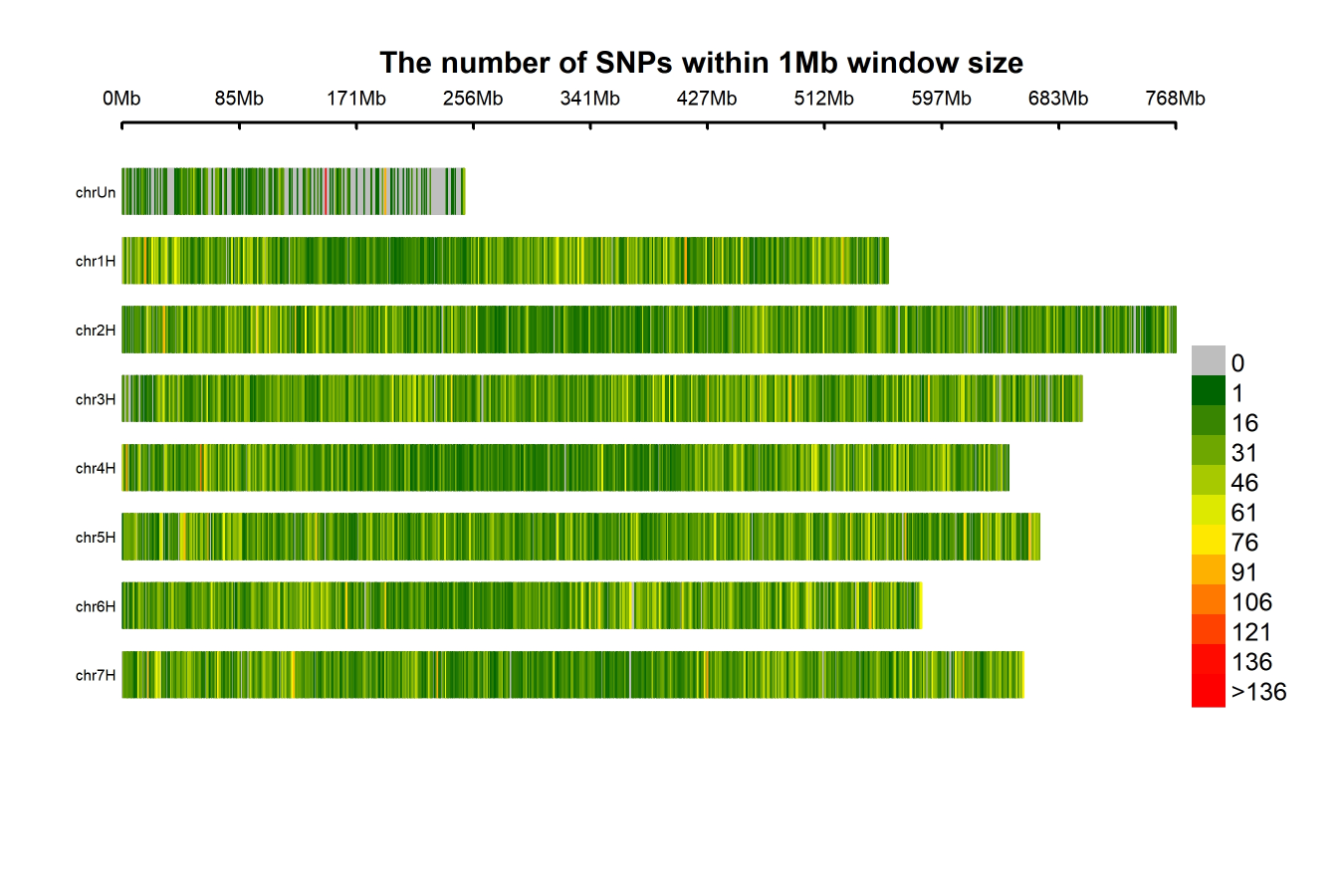


Figure S1 Distribution of 106,131 SNPs on the 7 chromosomes of barley.


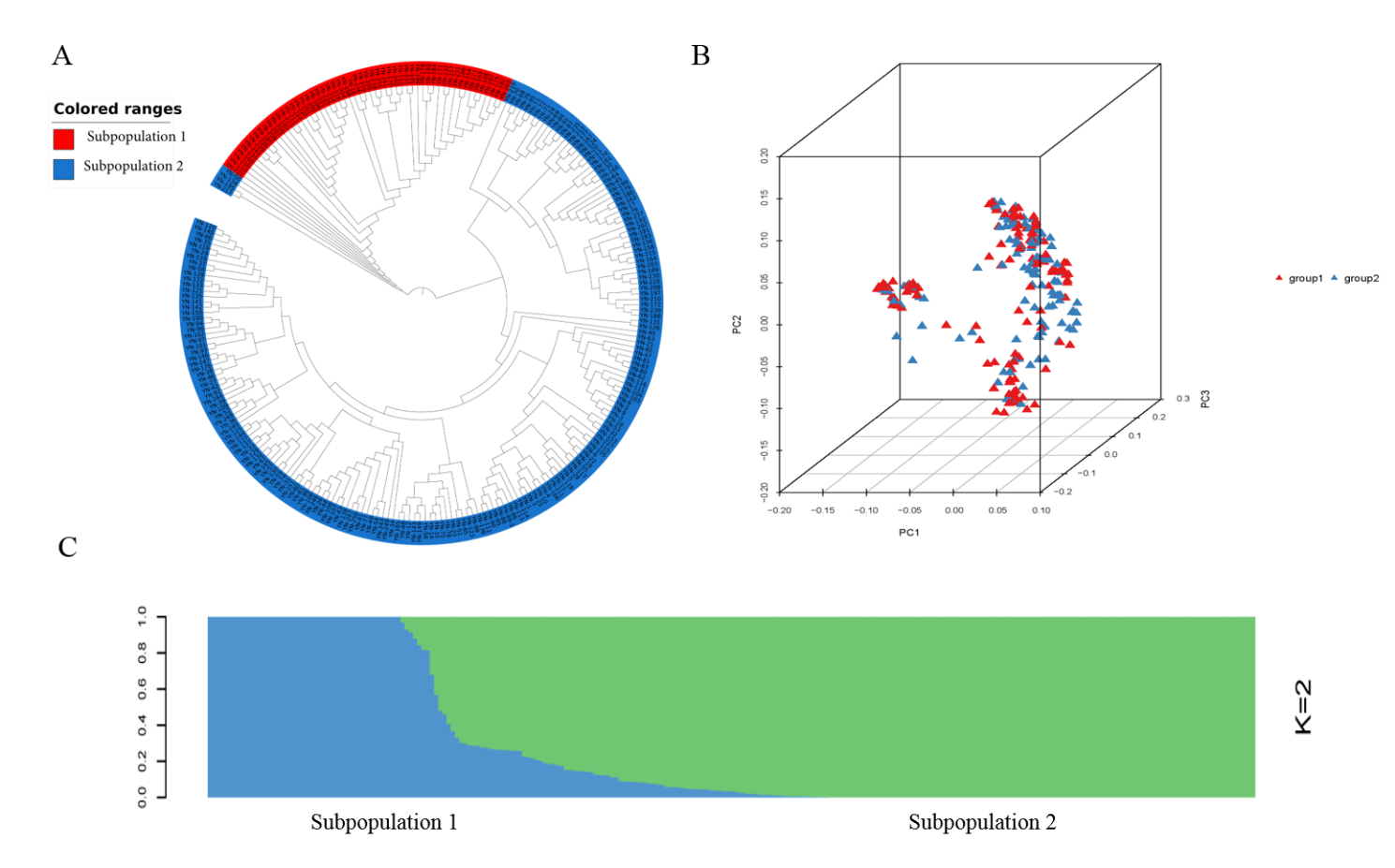


Figure S2 Population structure of the 250 accessions


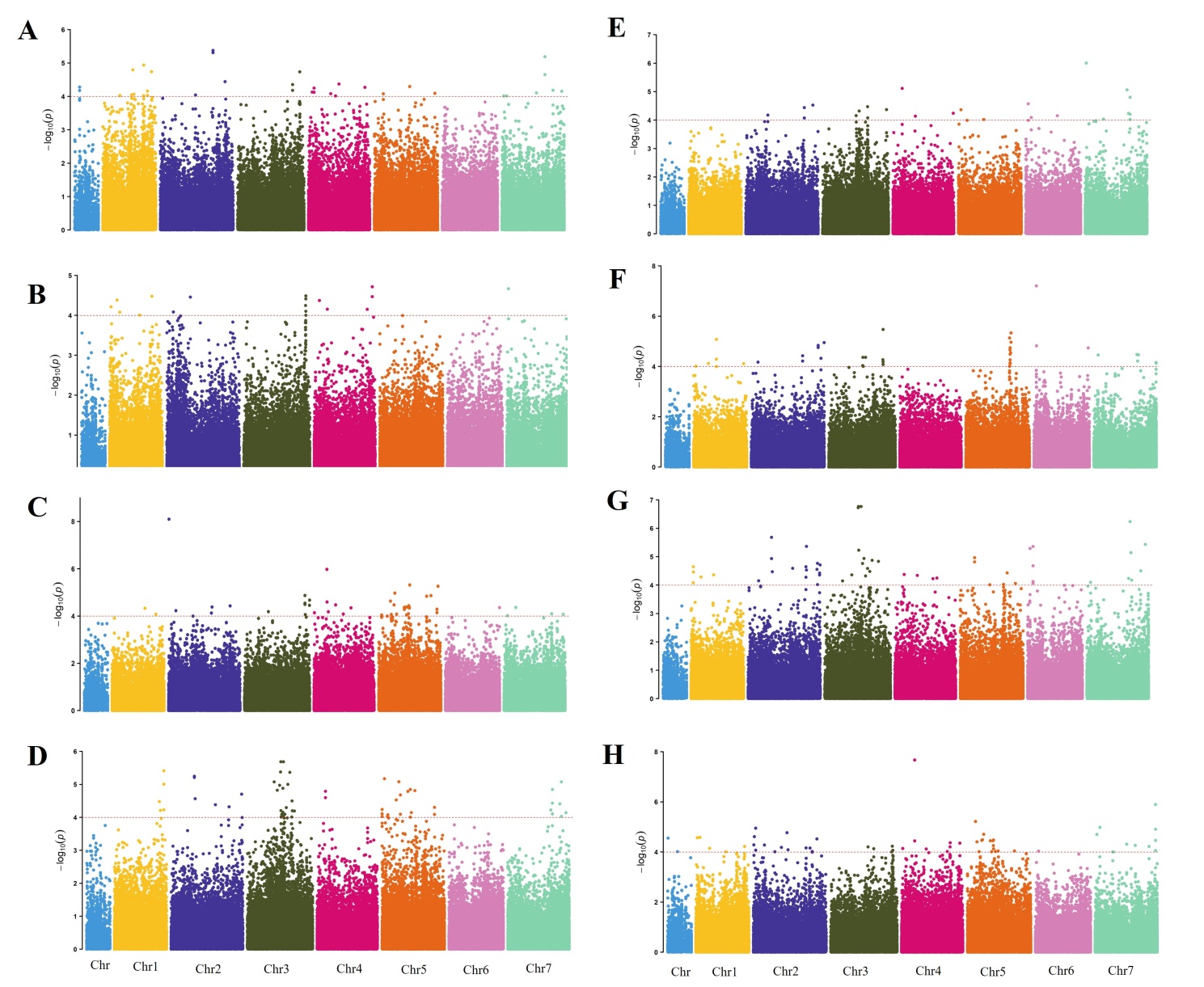


Figure S3 Manhattan plots resulting from the SNP-based GWAS in waterlogging treatment under different periods


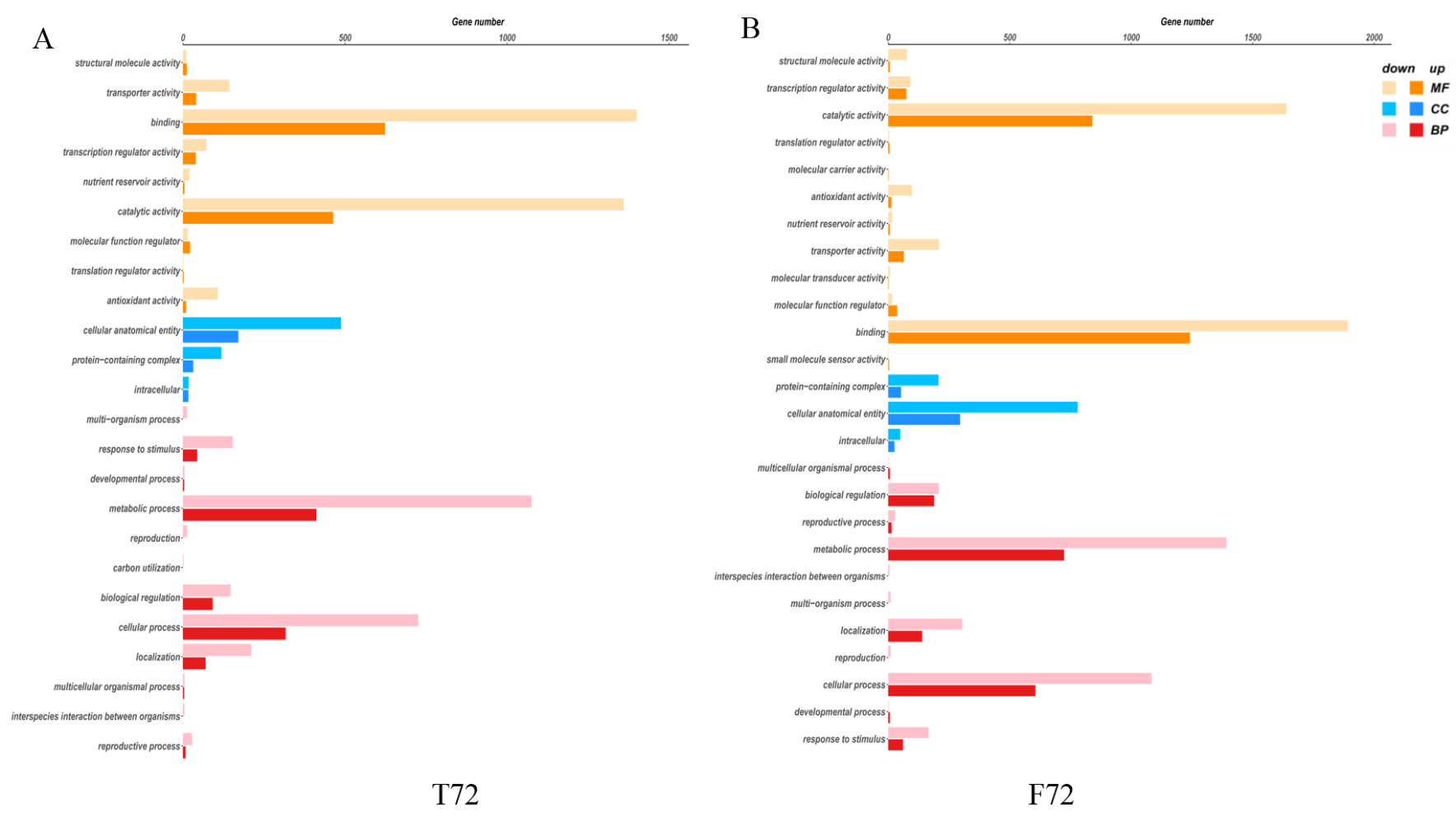


Figure S4 GO functional enrichment analysis of DEGs in the roots of two barley varieties under the waterlogging and control treatments

Supplemental Data 1: Protein sequences for phylogenetic and cluster analysis of ERF TFs with conserved N-terminal motif (MCGGAI/L) in *Arabidopsis* and barley.

| **HRE1** |
| --- |
| MCGGAVISDYIAPEKIARSSGKSSWRSNGVFDCSIYDFDGNFDELESDEPFVFSSTHKHHASGSASDGKKKQSSRYKGIRRRPWGRWAAEIRDPIKGVRVWLGTFNTAEEAARAYDLEAKRIRGAKAKLNFPNESSGKRKAKAKTVQQVEENHEADLDVAVVSSAPSSSCLDFLWEENNPDTLLIDTQWLEDIIMGDANKKHEPNDSEEANNVDASLLSEELLAFENQTEYFSQMPFTEGNCDSSTSLSSLFDGGNDMGLWS |

| **HRE2** |
| --- |
| MCGGAIISDFIWSKSESEPSQLGSVSSRKKRKPVSVSEERDGKRERKNLYRGIRQRPWGKWAAEIRDPSKGVRVWLGTFKTADEAARAYDVAAIKIRGRKAKLNFPNTQVEEEADTKPGGNQNELISENQVESLSEDLMALEDYMRFYQIPVADDQSATDIGNLWSYQDSN |
| **RAP2.12** |
| MCGGAIISDFIPPPRSRRVTSEFIWPDLKKNLKGSKKSSKNRSNFFDFDAEFEADFQGFKDDSSIDCDDDFDVGDVFADVKPFVFTSTPKPAVSAAAEGSVFGKKVTGLDGDAEKSANRKRKNQYRGIRQRPWGKWAAEIRDPREGARIWLGTFKTAEEAARAYDAAARRIRGSKAKVNFPEENMKANSQKRSVKANLQKPVAKPNPNPSPALVQNSNISFENMCFMEEKHQVSNNNNNQFGMTNSVDAGCNGYQYFSSDQGSNSFDCSEFGWSDQAPITPDISSAVINNNNSALFFEEANPAKKLKSMDFETPYNNTEWDASLDFLNEDAVTTQDNGANPMDLWSIDEIHSMIGGVF |
| **RAP2.2** |
| MCGGAIISDFIPPPRSLRVTNEFIWPDLKNKVKASKKRSNKRSDFFDLDDDFEADFQGFKDDSAFDCEDDDDVFVNVKPFVFTATTKPVASAFVSTGIYLVGSAYAKKTVESAEQAEKSSKRKRKNQYRGIRQRPWGKWAAEIRDPRKGSREWLGTFDTAEEAARAYDAAARRIRGTKAKVNFPEEKNPSVVSQKRPSAKTNNLQKSVAKPNKSVTLVQQPTHLSQQYCNNSFDNSFGDMSFMEEKPQMYNNQFGLTNSFDAGGNNGYQYFSSDQGSNSFDCSEFGWSDHGPKTPEISSMLVNNNEASFVEETNAAKKLKPNSDESDDLMAYLDNALWDTPLEVEAMLGADAGAVTQEEENPVELWSLDEINFMLEGDF |

| **RAP2.3** |
| --- |
| MCGGAIISDYAPLVTKAKGRKLTAEELWSELDASAADDFWGFYSTSKLHPTNQVNVKEEAVKKEQATEPGKRRKRKNVYRGIRKRPWGKWAAEIRDPRKGVRVWLGTFNTAEEAAMAYDVAAKQIRGDKAKLNFPDLHHPPPPNYTPPPSSPRSTDQPPAKKVCVVSQSESELSQPSFPVECIGFGNGDEFQNLSYGFEPDYDLKQQISSLESFLELDGNTAEQPSQLDESVSEVDMWMLDDVIASYE |

| **HORVU5Hr1G062940** |
| --- |
| MCGGAILAGFIPPSAAAAAAKAAATAKKKQQQRSVTADSLWTGLRKKADEEDFEADFRDFERDSSEEEDDEVEEVPPPPAPATAGFAFAAAAEVALRAPARRDAAVQHDGPAAKQVKRVRKNQYRGIRQRPWGKWAAEIRDPSKGVRVWLGTYDTAEEAARAYDAEARKIRGKKAKVNFPEDAPTVQKSTLKPTAAKSAKLAPPPKACEDQPFNHLSRGDNDLFAMFAFSDKKVPAKPTDSVDSLLPVKHLAPTEAFGMNMLSDQSSNSFGSTDFGWDDEAMTPDYTSVFVPSAAAMPAYGEPAYLQGGAPKRMRNNFGVAVLPQGNGAQDIPAFDNEVKYSLPYVESSSDGSMDNLLLNGAMQDGASSGDLWSLDELFMAAGGY |
| **HORVU5Hr1G080790** |
| MCGGAIIYDYIPAAAHRRRASTADFWHDANDHSDAYSAAPDKAPRAKRGRKNQYRGIRQRPWGKWAAEIRDPVKGVRVWLGTYPTAEAAARAYDRAARRIRGAKAKVNFPNEILAGAPAHQASCTMAAAPPPAALPSPKKEEGVEPAAPCSCEEVKALSEELMAYESYMSFLGVPYMEGGAAAAVGAAAEEAPAELWSFEDSYYYPGPLGL |
| **HORVU1Hr1G058940** |
| MCGGAVIADFVPAAARRPDGSSTDVPGSSLTGEEVTEKAPAPARKTAYRGIRRRPWGRWAAEIRDPRKGARVWLGTYATAEEAARAYDVAARDIRGVKAKLNFPPAVGAPQAAVAGAPKKRPRVAAEESSASWSPLPGTATGVGSTDSLRESMSGLEAFLGLKDAAGDDDVQPWEAVDIIF |
| **HORVU4Hr1G077310** |
| MCGGAILAELISPSAGRAPKQAQVAAAGPVSANKDGKSKGHKHNKYGSVADVDDDLQDFDDDLDLRQAEEDADDHVVFACKPAFSPGPAYDGGRAAQAASRKKKRVLHGIRQRPWGKWAAEIRDPHKGTRVWLGTFDTADDAARAYDVAARRLRGSKAKVNFPDAARAGARPRRASRRTAQKPQRPPAWTTAYSATAAAHSQPEQDAFVDLTTAVTALPPIMESSFADSGSTKPMFHEDSSAGSGGGAMPGFTDELGFDPFMLFQLPCSDTYESIDSLFAGDAALQDARGVDSGIDGVSLWSFDEFPMDSAIF |

Supplemental Data 2: CDS sequence of *HvERF1* in TX9425

HvERF1 of TX9425

ATGTGGGGCGGCGCCATCATCTACATACTACCTCTCGGCGGCGGAACACCGCCGCCGGGCGTCCACCGCCGACTTCTGGCACGACGCCAACGACCACTCCGACGCCTACAGCGCCGCCCCCGACAAAGCGCCGCGCGCGAAGCGGGGGAGGAAGAACCAGTACCGCGGCATCCGGCAGCGGCCGTGGGGGAAGTGGGCGGCGGAGATCCGCGACCCCGTCAAGGGCGTCCGCGTCTGGCTCGGCACCTACCCCACCGCCGAGGCCGCCGCGCGCGCCTACGACCGCGCCGCGCGCCGCATCAGGGGCGCCAAGGCCAAGGTCAACTTCCCCAACGAGATCCTCGCCGGCGCGCCCGCGCACCAGGCGTCGTGCACGATGGCGGCCGCGCCGCCGCCGGCCGCGCTCCCTTCCCCCAAGAAAGAGGAGGGGGTCGAGCCCGCGGCGCCGTGCTCCTGCGAGGAGGTGAAGGCGCTGTCCGAGGAGCTGATGGCGTACGAGAGCTACATGAGCTTCCTCGGCGTCCCCTACATGGAGGGCGGGGCCGCGGCCGCCGTCGGTGCCGCCGCCGAGGAGGCGCCGGCCGAGCTTTGGAGCTTCGAGGACAGCTACTACTACCCGGGGCCTCTGGGGCTCTGATCATCGTACCGTGCCCGTGCTGGCCGCGGCGAACTGAGTCAATTCCGTTGAATTCATTTTTTTCAGTTTTGTAACCGGTGA
